# Cervical Cancer Detection Using Deep Neural Network and Hybrid Waterwheel Plant Optimization Algorithm

**DOI:** 10.3390/bioengineering12050478

**Published:** 2025-04-30

**Authors:** Sarah A. Alzakari, Amel Ali Alhussan, S.K. Towfek, Marwa Metwally, Dina Ahmed Salem

**Affiliations:** 1 Department of Computer Sciences, College of Computer and Information Sciences, Princess Nourah bint Abdulrahman University, P.O. Box 84428, Riyadh 11671, Saudi Arabia; 2 Computer Science and Intelligent Systems Research Center, Blacksburg, VA 24060, USA; 3 Applied Science Research Center, Applied Science Private University, Amman, Jordan; 4 Jadara University Research Center, Jadara University, Irbid 21110, Jordan; 5 Faculty of Engineering, Misr University for Science and Technology (MUST), 6th October City 12585, Egypt

**Keywords:** classification, cervical, cancer, machine learning, waterwheel plant algorithm, particle swarm optimization algorithm, medical diagnosis

## Abstract

More than 85% of the world’s cervical cancer fatalities occur in less-developed nations, causing early mortality among women. In this paper, we propose a novel approach for the early classification of cervical cancer based on a new feature selection algorithm and classification method. The new feature selection algorithm is based on a hybrid of the Waterwheel Plant Algorithm and Particle Swarm Optimization algorithms, and bWWPAPSO denotes it. Meanwhile, the new classification method is based on optimizing the parameters of a multilayer perceptron neural network (WWPAPSO+MLP). A publicly available dataset is employed to verify the effectiveness of the proposed approach. Due to this dataset’s imbalance and missing values, it is preprocessed and balanced using SMOTETomek, where undersampling and oversampling were utilized. The usefulness of class imbalance and feature selection based on the classifier’s specificity, sensitivity, and accuracy has been demonstrated by way of a comparative study of the proposed methodology that has been carried out. WWPAPSO+MLP achieves superior performance, with an accuracy of 97.3% and a sensitivity of 98.8%. On the other hand, several statistical tests were conducted, including the Wilcoxon signed rank test and analysis of variance (ANOVA) to confirm the effectiveness and superiority of the proposed approach.

## 1. Introduction

According to the World Health Organization (WHO), the most common causes of untimely death among women across the world are malignancies of the female reproductive tract, including breast, cervical, and ovarian cancer [1]. Cervical cancer, also known as cancer of the cervix (the lowermost section of the uterus), is a malignant tumor that develops when tissue cells covering the cervix begin to expand and multiply uncontrolled without following the appropriate mechanism for cell division [2]. Cervical cancer is sometimes referred to as cancer of the uterine cervix. According to the figures that the WHO provides, more than 270,000 women pass away each year as a result of cervical cancer. More than 85% of these fatalities occur in poor countries, and it is projected that there are 444,500 new instances of cervical cancer each year [3]. There has been a notable rise in the number of people diagnosed with cervical cancer in developed nations like the United States and the United Kingdom [4]. An estimated population of 50.33 million women aged 15 years and older in the developing nation of Nigeria are in danger of acquiring cervical cancer. Cervical cancer is regarded as the second most prevalent malignant tumor among women in Nigeria, with a high death rate among those who are affected [5]. On the other hand, if it is caught in its early stages, it is frequently curable with the removal of the affected tissues [6]. Huge volumes of information on cancer have been compiled thanks to the development of innovative technology in the healthcare sector, and these records are now readily available to the scientific community [7]. Researchers in machine learning (ML) are continually working to build improved prediction models capable of analyzing the data currently available in the field of cervical cancer. The process of diagnosing cervical cancer can be sped up with the use of predictive models that were constructed using machine learning techniques [8]. Although these models can predict the outcomes of cervical cancer cases, there are several limitations of these models such as solving the problem of overfitting [9], resampling techniques in handling skewed data and less application of data balancing [10], and less application of dimensionality reduction techniques [11,12]. Nevertheless, these models are still able to predict the outcomes of cervical cancer cases. As a result, a novel strategy for the development of an ML model has the potential to give the possibility of combating cervical cancer in a manner that is more cyclopedic and creates a healthier future for girls and women.

Cervical cancer continues to pose a significant global health burden, particularly among women in developing countries, where access to regular screening and early intervention is limited [13]. The disease is the fourth most common cancer in women worldwide and is largely preventable through early detection and vaccination [14]. However, traditional screening methods such as the Pap smear and HPV testing often face barriers, including high costs, limited infrastructure, and patient non-compliance. Early diagnosis and clinical decision support systems developed from data solutions must address populations with limited access to care because these underserved populations require these systems the most.

Machine learning and deep learning methods demonstrate significant potential for automated cervical cancer diagnosis through demographic and clinical datasets according to research done in the past few years [15]. A scarcity of studies presents statistical evaluation techniques while failing to perform cross-setting comparisons in their findings [16]. The research presents a robust framework that unites an MLP classifier with a newly designed bWWPAPSO algorithm that implements binary hybrid feature selection through the Waterwheel Plant Algorithm and Particle Swarm Optimization. The proposed algorithm targets three objectives: improving prediction performance, emphasizing essential features, and achieving strong performance across new unseen information.

This research proposes a new approach for predicting cervical cancer given the preliminary resulting of screening from individual medical records. The proposed prediction model would help in the intelligent detection of cervical cancer using deep learning and swarm optimization approaches. In addition to supporting the diagnosis of cervical cancer, the model that has been developed may also be utilized in actual medical facilities to support the diagnosis of other forms of cancer, including breast, prostate, and blood cancers. Predicting the results of biopsies performed on cervical cancer patients is the primary subject of this article. To evaluate the performance of the model, it provides a stronger emphasis on having a better positive value of the outcome of the cervical cancer test; accordingly, it concentrates more on sensitivity rather than calculating other metrics such as accuracy at the same time. The reason for this is that it has a primary emphasis on achieving accurate findings for patients who are afflicted with the condition, which means that it strives to get a higher positive value result. The paper’s significant findings can be summarized and expanded upon as follows:

- A novel hybrid optimization approach is proposed to optimize the parameters of the proposed prediction model based on the waterwheel plant and Particle Swarm Optimization algorithms.
- The results achieved by the proposed deep neural network are compared to the results of state-of-the-art prediction models.
- The results of the optimized neural network are compared to three other optimization algorithms to prove the superiority of the proposed algorithm.
- A set of statistical tests is performed to study the statistical difference, stability, and significance of the results achieved by the proposed approach compared to a set of other competing approaches.

The structure of this paper includes the following sections. Section 1 is the introduction of the topic addressed in this paper. Section 2 addresses the literature review to shed light on the previous works related to cervical cancer using artificial intelligence. In Section 3, the proposed methodology is explained. In Section 4, a summary of the proposed research findings is presented and discussed. Section 5 of this paper includes the summary and concluding results of the achieved results.

## 2. Literature Review

In this section, several publications attempting to categorize cervical cancer are presented and discussed.

### 2.1. Feature Selection

It does this by picking a subset of features that make a significant contribution to the target class. This, in turn, leads to an increase in the overall predictive capacity of the classifier [17], as well as a reduction in the length of the whole process and the computational cost [18]. When it comes to lowering the dimensionality of the data, the selection of features is the key strategy that is utilized [19]. It has been demonstrated in reference [18] that the performance of ML approaches improves as the dimension of the features being analyzed is decreased. When a dataset is provided with a collection of characteristics as input, dimensionality can be reduced by picking the best possible subset of those features from the dataset [20]. In addition, reducing a problem’s dimensionality helps eliminate unnecessary features, cut down on noise, and provide for a more trustworthy learning model because fewer features are involved. Feature selection is reducing the dimensionality of a dataset by choosing new features that are a subset of the existing ones [21].

#### 2.1.1. Embedded Methods

Several researchers have developed hybrid feature selection strategies [22]. These techniques combine the strengths of the two methods that were previously discussed. To pick the most appropriate feature subset, the embedded approach incorporates both the filter method and the wrapper technique [23]. It is computationally less expensive than the wrapper approach, more accurate than the filter technique, and considers all of the features simultaneously [24]. Although it chooses features unique to the model, it has the following benefits over both methods: it selects features that are particular to the model. LASSO is the name of a technique that is frequently employed in embedded feature selection. It was initially presented by [25] for parameter estimation and also for the selection of features. The penalized least squares regression with the L1-penalty function is the general framework upon which LASSO is built. LASSO offers a method for effective feature selection that is predicated on the premise that there is a linear dependence between the characteristics that are input and the output that is desired [26]. This method has seen extensive use in classification problems, specifically to choose the best feature subset. The L1 regularization causes LASSO to reduce the absolute sum of the coefficients. An ML model for predicting individuals who suffer from cardiovascular disorders was created in reference [27]. Two embedding methodologies, namely, the relief and LASSO procedures, were utilized in the selection process for pertinent characteristics. According to the analysis of the results, the suggested model achieved an accuracy of 97.65% while using the LASSO feature selection approach in conjunction with the Random Forest Bagging Method (RFBM). This result was superior to the one obtained without LASSO, which was 92.65%. The LASSO algorithm was used to predict spot prices for energy to pick attributes that were important to the task. Because it can identify the most accurate attributes for the prediction of spot prices for energy, this method works very well for them in their forecast. The mean average prediction error was reduced by as much as 16.9% thanks to the improvements in prediction accuracy [28]. When it was put to use in real-world picture and biological feature selection tasks [26], LASSO delivered a result that was deemed to be very encouraging. An embedded technique of features election utilizing LASSO was also presented in the reference [29], which was applied to the input nodes of neural networks. The strategy that has been presented creates group sparsity and prunes weights in a grouped way, which ultimately results in the elimination of characteristics that are not helpful. The filter method performs an independent test without engaging any machine learning methodology. In contrast, the wrapper method calls for a specified machine-learning strategy to evaluate a subset of the features. In contrast to wrapper techniques, which often have a higher degree of classification accuracy but need a significant amount of computer power, filter approaches have minimal computational costs but inadequate dependability in classification. Filter approaches have a cheap computing cost, but they do not provide adequate reliability in classification. Wrapper methods, on the other hand, tend to complement each other in such a way that they tend to complement each other. The components of the filter method and the components of the wrapper techniques are combined in the embedded approach in order to achieve the goal of dimensionality reduction. In addition, to demonstrate the significance of feature selection within the scope of this article, a comparative analysis of the two different methodologies for selecting features (RFE and LASSO) was carried out.

#### 2.1.2. Wrapper Method

An ideal subset of characteristics is chosen in the model based on the inferences drawn from the model that came before it. Wrapper techniques can discover the interactions between the various aspects of the dataset, and their prediction performance is frequently superior to that of filter approaches. By training a model on a certain subset of features, this method determines how effective that subset of features is. As a result, the computation required by these approaches is more significant [2,22]. The RFE [30] is a frequently utilized method to select wrapper features for situations with tiny samples. The RFE accomplishes its goal by recursively removing attributes and then developing a model based on those left behind. It determines which attribute (and combination of attributes) contributes the most to predicting the target attribute(s) by using the accuracy of the model [30]. RFE tends to get rid of “weak” characteristics, which ultimately leads to creating a more accurate prediction model with fewer dimensions [31]. When there are fewer characteristics to consider, a classifier can better focus on creating a model that requires less run time [32]. RFE is used in a variety of diagnostic applications in the medical field. The RFE method was used to solve issues pertaining to gene selection for microarray data [33,34]. There might be thousands of characteristics in such data, but the writers would typically eliminate half of them before moving on to the next phase. Because it deletes features in a recursive manner utilizing feature weight coefficients (for example, linear models) or feature significance (tree-based algorithms), RFE is computationally easier than SFS. However, SFS is more complex. SFS, on the other hand, is able to remove (or add) features at the discretion of a user-defined classifier/regression performance parameter [35]. By providing an explanation of the significance of data cleaning, the replacement of missing values, and the use of feature selection procedures, the reference [2] illustrated the significance of model creation. This was accomplished in order to attain efficacy in outcome prediction with an optimal feature subset. For the purpose of determining the most important risk factors for cervical cancer detection, they evaluated a number of different feature selection approaches, such as the simulated algorithm (SA), the Boruta algorithm, and the recurrent feature expansion (RFE). Regarding the cervical cancer prediction method, reference [36] utilized RFE in order to ascertain the elements that have a substantial impact on the process. LR, multilayer perceptron, and SVM classifiers were used, and their performance was assessed according to accuracy, specificity, and area under the curve (AUC). The SVM that contained the detected characteristics performed better than others, with an accuracy of 91.04%, specificity of 91.94%, and area under the curve (AUC) of 89%, respectively.

#### 2.1.3. Filter Method

Statistical analysis is utilized in the filter technique to pick the characteristics to use in the algorithm depending on how closely they are related to the variable that is the focus of the analysis. Most of the time, filtering techniques are utilized prior to classification to exclude factors that are not very important [21]. They are a vast improvement over all previous feature selection strategies in terms of speed and the amount of computer resources required [37]. The drawback of the approach is that it does not interact with other characteristics, and it does not consider the model being used. Table 1 summarizes the feature selection methods.

### 2.2. Class Balancing

The classification categories in a dataset have an unbalanced representation if they are not all present in equal numbers. In many cases, the “normal” samples make up the vast majority of the real-world datasets, while the “abnormal” or “interesting” examples make up only a small portion of the total. This imbalance gives birth to the “class imbalance” problem [38], which is the challenge of understanding a concept from a group that only has a limited amount of observations. Research has demonstrated that having balanced data may lead to higher prediction performance. As a result, various well-known strategies have been developed and employed in machine learning to solve this issue and improve the prediction models’ performance [18]. The problem of class imbalance has been encountered in a variety of domains, including medical diagnosis/monitoring, fraud/intrusion detection, bioinformatics, text classification, and telecommunications management, and it is considered to be one of the top 10 difficulties associated with data mining [39,40]. When imbalanced information is present, the learning process is significantly impacted since most standard machine learning algorithms anticipate a balanced class distribution [41]. For this reason, many methods tailored to the management of big datasets have been developed and put into practice. To address the issue of an unevenly distributed dataset, there are fundamentally three distinct approaches that may be taken: (i) undersampling, (ii) oversampling, and (iii) hybrid undersampling and oversampling.

An approach known as undersampling primarily aims to preserve the existing class distribution by systematically excluding classes from which they are more likely to be represented. In the process of undersampling, the number of samples taken from the majority class is decreased until they are on par with the number of samples taken from the minority class [40]. Several different research projects have resulted in the development of hybrid sampling approaches, which mix oversampling and undersampling to provide a dataset that is representative of the whole population. The SMOTETomek method is a good illustration of such a strategy. The synthetic minority oversampling technique (SMOTE) is an example of an oversampling method, while the Tomek approach is an example of an undersampling method. Together, these two methods make up the hybrid approach known as Tomek. For SMOTE to function, each minority class’s sample is first isolated, and then synthetic samples are introduced along the line segments that connect any/all of the k minority class’s nearest neighbors [42]. Tomek linkages identified an issue with noise and borderline sampling when the undersampling approach was applied. Undersampling is another term that may be used to describe the Tomek Links methodology. They may be recognized as a pair of classes most unlike each other’s nearest neighbors since the distance between them is the shortest [43]. They are put to use in the process of removing overlapping samples that are added by SMOTE [44].

### 2.3. Classification of Cervical Cancer

In recent years, data have been collected and are currently in a state where the community of medical researchers may easily access them [3]. Several efforts have been made to employ ML in activities such as prediction to assist in the early identification of cervical cancer [45]. Therefore, earlier research on the Risk Factors dataset, which may be accessed without charge in the University of California, Irvine (UCI) repository, serves as the primary impetus for our study [46]. In the study referred to as reference [11], an upgraded Decision Tree (DT) classifier was utilized on the Risk Factors dataset to classify individuals diagnosed with cervical cancer. The DT classifier presented is utilized to classify patients diagnosed with cervical cancer; nevertheless, there was no application of a feature selection strategy to pick an ideal subset of features. The dataset, including cervical cancer risk variables, was evaluated by the reference [47] utilizing three different Support Vector Machine (SVM)-based methods. In reference [9], many machine learning (ML) classifiers, including Gaussian naive Bayes (GNB), Discriminant Trees (DT), Logistic Regression (LR), K-Nearest Neighbors (KNN), and Support Vector Machines (SVM), are investigated to classify individuals who have cervical cancer. It was clear that the DT classifier had performed far better than the other classifiers since its accuracy was 97%. Overfitting the model is a flaw in their research that has to be addressed. They separated the dataset using the hold-out strategy, which was appropriate given the small number of observations (858). The k-fold cross-validation technique with [48] a significant value of k is the approach that should be used when working with small datasets. This method will permit all datasets to participate in both the training and testing, offering a more accurate result than the hold-out method [49]. The SVM, Random Forest, and Gradient Boosting machine (GBM) models were combined using the synthetic minority oversampling method (SMOTE) in reference [8]. A genetic algorithm (GA) was used for feature selection, and Bayesian optimization was used for hyperparameter tuning.

Based on sensitivity, a comparison research project including all of the models was carried out. Their findings demonstrated that GBM has the highest sensitivity, at 77.8%, followed by SVM, which had a sensitivity of 55.58%, and Random Forest, which had a sensitivity of 44.4%; hence, their findings have the potential to be improved to reach good model performance. A DT classifier was built by reference [10] to predict individuals diagnosed with cervical cancer. There was no strategy to deal with the disparity in social status. Classifier decision boundaries can be skewed in favor of the majority class when models are trained using unbalanced datasets. Consequently, it is of the utmost importance to successfully tackle class imbalance by employing preprocessing approaches such as oversampling the class of the minority or undersampling the class of the majority. For cervical cancer prediction, the research cited in reference [12] utilized ML approaches such as boosted Decision Trees, decision forests, and decision jungle algorithms. The boosted Decision Tree fared better than the other approaches, with an AuC curve of 97%. SMOTE was utilized to overcome the issue of data imbalance; nevertheless, their approach did not address the problem of redundant features by employing a feature selection strategy to lower the problem’s dimensionality. A classification model for cervical cancer was developed in reference [50] by utilizing RF with SMOTE in conjunction with two feature reduction approaches, namely, RFE and principal component analysis (PCA). Their findings revealed that the combination of RF and SMOTE has a comparatively superior performance, with an accuracy of 96% in predicting the result of the patient’s biopsy. This was determined after comparing the data obtained. The dataset of risk factors was examined using Support Vector Machines (SVM), Extreme Gradient Boosting (XGBoost), and Random Forests (RF) in reference [51]. Before the categorization system, the class imbalance problem was addressed with SMOTE. According to the findings of their classification, XGBoost and Random Forest perform significantly better than SVM when predicting the biopsy outcome, with sensitivity levels of 94% and 95%, respectively. A voting approach incorporating the DT, LR, and RF classifiers was utilized in constructing a classification model for cervical cancer that was cited in reference [52]. PCA was employed as a technique to help minimize features, while SMOTE was utilized to help tackle the problem of an imbalanced dataset. According to their findings, the voting classifier, SMOTE, and PCA approach improved the prediction model’s accuracy, sensitivity, and area under the curve (AUC). Accuracy, sensitivity, and positive predictive ability (PPA) ratios significantly increased inside the SMOTE voting model, going from 0.93% to 5.13%, 39.26% to 46.97%, and 2% to 29%, respectively.

The fact that the SMOTE approach generates synthetic samples, which causes classifiers to build decision areas that are bigger and less particular rather than smaller and more specific, is the technique’s most significant shortcoming [53]. A voting mechanism was utilized in the ensemble approach described in reference [54], which was used to estimate the probability of developing cervical cancer. Although the ensemble technique overcomes the difficulties present in the earlier research on cervical cancer, the sensitivity rate is relatively low, which can be related to the unbalanced datasets. Extreme learning machines (ELMs) and convolutional neural networks (CNNs) were used to construct a model for the categorization of cervical cancer that was published in reference [55]. The writers accessed the information from the Herlev database. The accuracy of the proposed CNN-ELM-based system proposed was 99.5% when applied to the issue of predicting two classes, and it was 91.2% when applied to classifying seven classes. Although neural networks have been utilized in several different classification tasks [56], they are time-consuming and call for massive datasets. A neural network is a technology known as a black box because it can reduce the amount of room for interpretability. A cervical cancer prediction model (CCPM) was developed as a consequence of the study that was referenced in reference [57]. This model is capable of providing an early prediction of cervical cancer by utilizing a number of risk indicators. Both density-based spatial clustering of applications with noise (DBSCAN) and isolation forest (iForest) are among the outlier detection methods that are utilized by the CCPM in order to eliminate outliers. Both of these strategies are designed to identify and isolate data points that are statistically significant as being outside the norm. They used SMOTETomek and SMOTE to resample the dataset, including cervical cancer risk variables. In the end, they used a Random Forest called RF as the basis classifier. Table 2 summarizes the studies related to classification of cervical cancer.

Multiple current research works highlight how machine learning methods improve both early diagnosis methods and survival rate assessments for cervical cancer patients. The research published in [13] analyzes survival prediction for cervical cancer patients by machine learning methods, which reveals the significant function of time-to-event analysis for clinical decision support. The integration of this capability expands the potential of ML applications for cervical cancer by moving from diagnostic to prognostic applications.

A different research effort used classic machine learning models including the Support Vector Machine (SVM) and Gradient Boosting with Decision Tree (DT) and Adaptive Boosting and Random Forest (RF) to predict cervical cancer as described in [14]. Briefly described were findings that showed excellent classification success rates reaching 100% for multiple classifiers and used a user study consisting of 132 Saudi Arabian participants to gauge how the public receives AI for medical diagnostic purposes.

The authors of this paper [15] presented a federated machine learning system with IoMT and fuzzed neuron alignment and blockchain to ensure privacy for cervical cancer prediction. Their system reached 99.26% accuracy, proving secure distributed models for clinical applications while simultaneously strengthening both prediction performance and defense mechanisms for the model.

The authors of [16] established a predictive solution by uniting SVM with Gradient Boosting alongside DT and XGBoost through KNN methods. The research confirmed that hybrid classifiers show excellent performance capabilities with 96% accuracy accompanied by 98% precision together with recall and an F1-score. The paper encourages multidisciplinary teamwork for future development and promotes integration between emerging technologies and better datasets.

Researchers published recent findings about a deep convolutional neural network (CNN) framework that produced exceptional results in robust skin cancer classification and analysis on dermoscopic images according to study findings presented in [58]. The predictive task’s domain remains distinct, yet the challenges concerning class imbalance, deep feature learning, and model predictability match cervical cancer detection requirements. The research adopted augmentation alongside cross-validation techniques for improving model performance, which corresponded to our preprocessing pipeline methods. The research demonstrates that deep learning remains essential for medical classification duties through its combination with optimization and regularization techniques in neural networks. Studies in cervical cancer diagnosis and other diseases based on prediction use CNN models, ensemble methods, and federated learning approaches. The proposed research implements privacy-protecting hardware infrastructure that permits distributed clinical information processing while utilizing a combination of SVM and KNN and boosting technology-based classifiers. The proposed method combines a binary hybrid metaheuristic for feature selection and a deep MLP classifier, enabling an optimized approach to dimensionality reduction and enhanced generalization on imbalanced datasets.

### 2.4. Research Gaps and Contributions

Research difficulties remain prominent in the literature about using machine learning and nature-inspired optimization techniques to predict cervical cancer, even though scientific inquiry into this approach increases. The solution to these research gaps proves essential to enhance the diagnostic precision, interpretability, and generalization of predictive models when used in actual healthcare facilities.

- Inadequate Handling of Class Imbalance: Many earlier studies failed to address the class imbalance problem within cervical cancer datasets because the non-cancerous cases outnumber cancerous cancer cases. The deceptive accuracy statistics of biased classifiers arise from unbalanced classes, which causes them to recognize genuine cancer cases properly.
- Suboptimal and Isolated Feature Selection Strategies: Choosing features is a vital data simplification technique to enhance model effectiveness because feature selection directly affects model efficiency. Most current works choose single-method feature selection approaches while needing hybrid methods that exactly balance exploration and exploitation to discover the most critical features.
- Limited Adoption of Hybrid Optimization Algorithms: The adoption of hybrid algorithms that merge complementary search strategies stands as a rare occurrence because numerous studies continue to rely on metaheuristic optimizers like Genetic Algorithm (GA), Particle Swarm Optimization (PSO), or Whale Optimization Algorithm (WOA).
- Insufficient Statistical Validation: The evaluation of many studies depends entirely on fundamental accuracy and precision results because they do not perform actual statistical significance evaluations (e.g., ANOVA and Wilcoxon signed-rank) to establish the robustness of their results.
- Generic and Untuned Classifier Architectures: The implementation of generic classifier architectures together with untuned architecture components remains prevalent throughout existing works. Cervical cancer data analysis suffers when off-the-shelf models operate without proper adjustment since such restricted use inhibits the classifier from identifying precise domain-specific patterns in the data.

This research presents a new framework that uses a feature selection approach based on the bWWPAPSO combination between the Waterwheel Plant Algorithm and Particle Swarm Optimization (bWWPAPSO) followed by an optimized Multilayer Perceptron (MLP) classifier. This study presents its main contributions through the following points:

1. The proposed novel hybrid feature selection method (bWWPAPSO) achieves effective feature subset discovery by uniting exploration and exploitation operation mechanisms.
2. Applying SMOTETomek as a hybrid resampling technique helps reduce class imbalance effects, improving the recognition of minority class instances.
3. We utilize the chosen features to optimize a Multilayer Perceptron (MLP) neural network that functions as a specific cervical cancer prediction architecture.
4. In its comprehensive assessment, this study employs multiple performance metrics for analysis, including Accuracy, Sensitivity, Specificity, Positive Predictive Value (PPV), Negative Predictive Value (NPV), and F-score.
5. The statistical importance of our work receives validation through both ANOVA (Analysis of Variance) and Wilcoxon signed-rank testing, which confirms the solid, stable and advanced state of our proposed method.

The goal of this study is to create a performance-based diagnostic system that combines statistical validation methods for early detection of cervical cancer through an advanced classification system.

## 3. The Proposed Methodology

**Figure 1:**
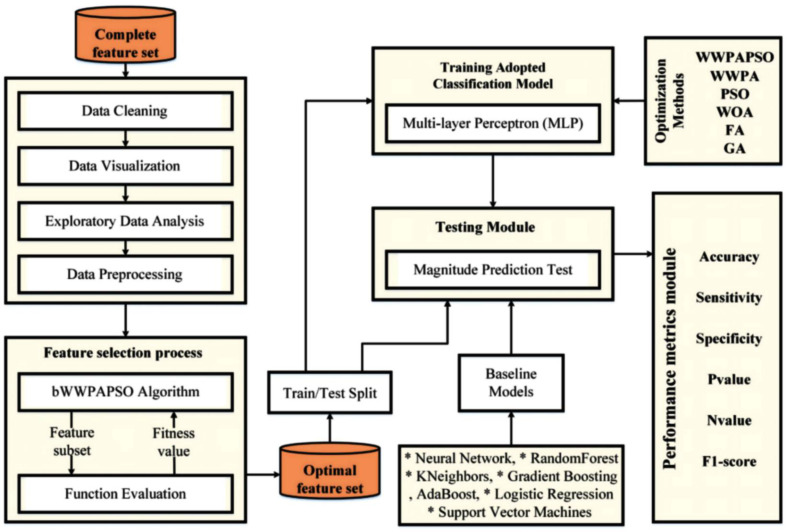
The architecture of the proposed methodology.

The fundamental architectural structure of the suggested model is depicted at the top of Figure 1. In addition, it demonstrated the structure of the model in terms of the numerous components that comprise the system and how these components are connected.

### 3.1. Dataset Description and Visualisation

**Figure 2:**
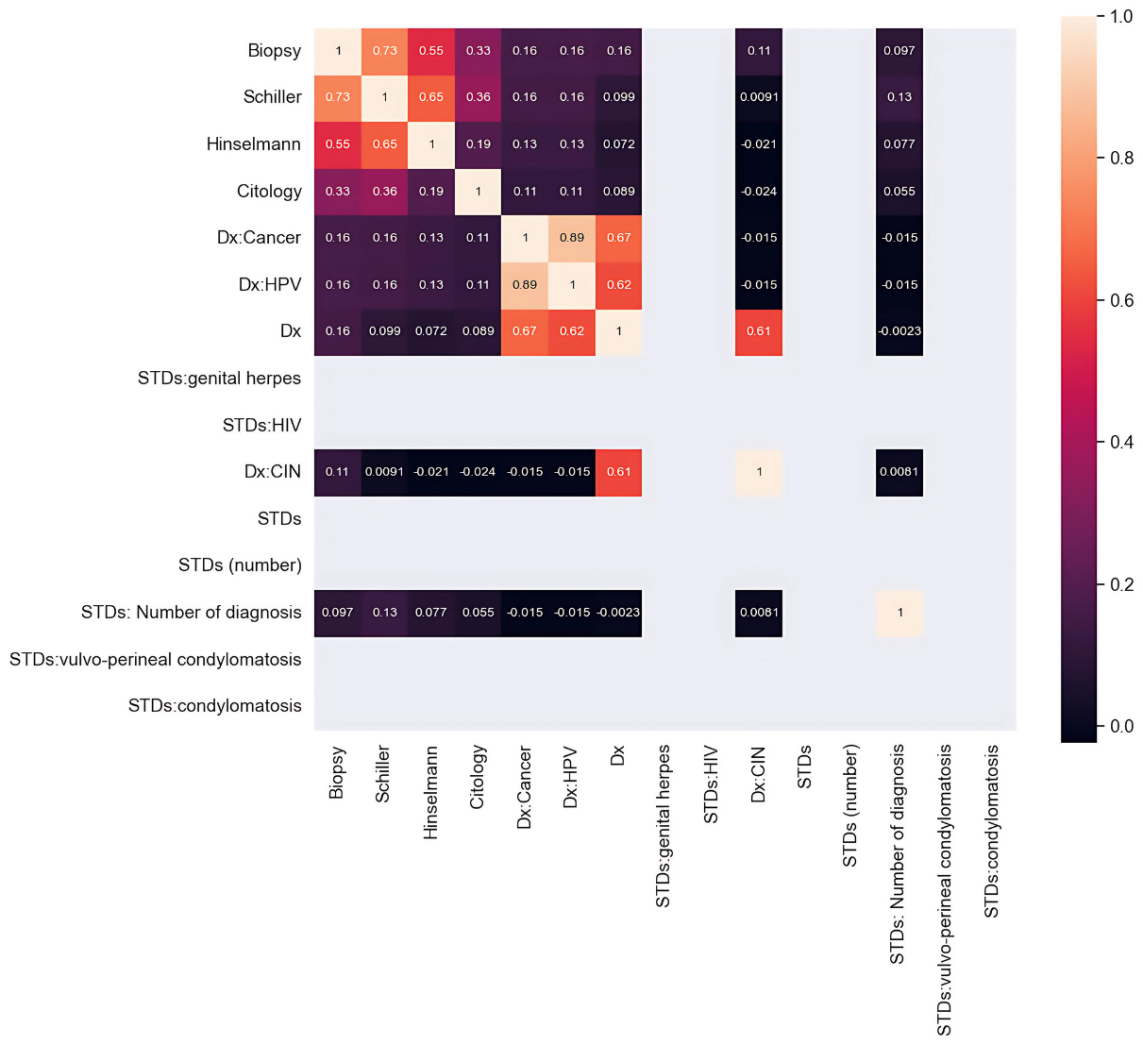
The heatmap of the features of the adopted dataset.

A hospital in Caracas, located in Venezuela, is where the data for the dataset was acquired. It is known as the Risk Factors dataset, and it may be found on the machine learning repository at the University of California, Irvine (UCI) [46]. This public dataset provides information on 858 patients (samples) along with 36 different features. The characteristics include medical histories, behavioral patterns, and demographic information. Unfortunately, the dataset has certain missing values; as a result, the dataset has to be processed to accommodate the missing values. These are the characteristics that are known to increase the likelihood of developing cervical cancer. The findings of the Hinselmann and Schiller diagnostic tests and cytology and biopsy, which are the most common diagnostic procedures for cervical cancer, will serve as the goal variables in this study. Due to an excessive number of missing values, the two characteristics “time since first diagnosis” and “time since last diagnosis” have been removed from the analysis to guarantee the reliability of our findings. The biopsy has traditionally served as the gold standard for identifying individuals suspected of having cervical cancer, and the purpose of this research is to develop a method that can anticipate the results of the biopsy. Figure 2 illustrates the heatmap of the features of the adopted dataset.

### 3.2. Data Preprocessing

The researchers utilized the Cervical Cancer Risk Factors dataset from the UCI Machine Learning Repository from a hospital in Caracas, Venezuela. The dataset consists of 858 records, which hold 36 different attributes that measure demographic characteristics and behavioral patterns and clinical test results such as Hinselmann and Schiller and Cytology and Biopsy. The sample data present continuous measures, including age together with several pregnancies and years of smoking and categorical measures that comprise HPV descriptions and HIV and STD diagnoses. The main goal of this research pertains to the ”Biopsy” variable as it detects whether the patient received a cervical cancer diagnosis. The Yes/No binary response functions as the fundamental truth for supervisor learning methods. Around 96% of the dataset entries match the non-cancerous class, whereas cancer-positive cases make up just about 4% of the entries. The class imbalance needs proper handling before progressing with generalization since SMOTETomek resampling was applied later. The available dataset lacks various values representing factors contributing to cervical cancer risks. We designed a thorough data preprocessing procedure for handling the issue. The processing of missing values involves two methods that are either removing instances or filling in the gaps. We removed all features that presented more than 60% of missing values from the data, including ”STDs: Time since first diagnosis” and ”STDs: Time since last diagnosis.” The remaining applications were processed through two data-handling methods. We removed complete records from the database; thus, our final sample size became 737 instances instead of 858. We used two methods to maintain the remaining data (1 and 2) by substituting numerical values with the mean and categorical values with the mode in a framework shown in Figure 1 and Figure 2.

(1) $$Mean\left(x\right)=\frac{1}{n}\left(\sum_{i=1}^{n}x_{i}\right)$$

where *i* is the ith value of variable *X*, $x_{i}$ is the ith variable, and *n* is the number of dataset variables.

(2) $$Model\left(Z\right)=L+\left(\frac{f_{m}+f_{m-1}}{\left(f_{m}-f_{m-1}\right)+\left(f_{m}-f_{m-1}\right)}\right)$$

where $f_{m-1}$ is the frequency of the preceding class, *m* represents the class mode, *L* is the lower boundary point of mode class, $f_{m}$ is the frequency of the mode class, *C* is the length of the mode class, and $f_{m+1}$ is the frequency of the succeeding class. It was also decided to exclude from the dataset the characteristics STDs_Time_since_last_diagnosis and STDs_Time_since_first_diagnosis that had a missing value percentage of more than sixty percent (787 out of 858).

### 3.3. Dataset Balancing Using SMOTE

**Figure 3:**
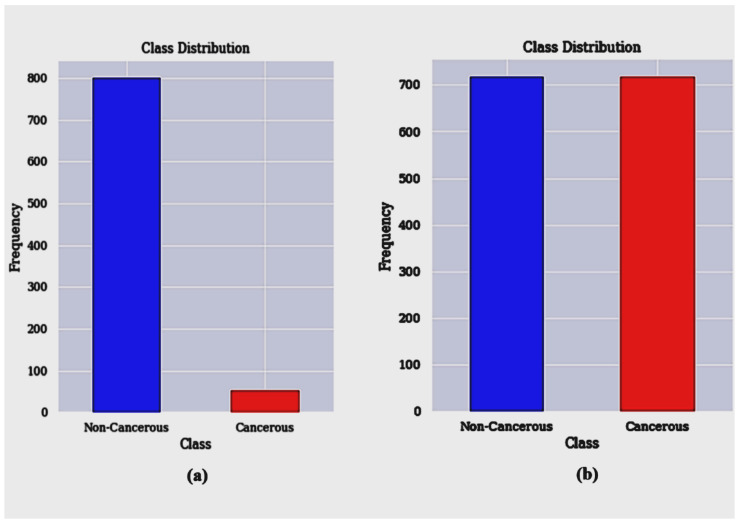
(**a**,**b**) The accuracy of cervical classification using the proposed methodology.

Due to the fact that 96% of the observations are non-cancerous instances and just 4% are malignant cases, the distribution of positive and negative classes in the dataset containing the risk factors for cervical cancer is severely skewed. This can be seen in Figure 1. The topic of unbalanced datasets in the cervical cancer risk factors dataset has received the least attention from previous publications. To rectify the significant data imbalance within the cervical cancer dataset, we use SMOTETomek. Undersampling (Tomek) and oversampling (SMOTE) are both components of the SMOTETomek resampling approach. Figure 3 illustrates the balanced classes that are produced as a consequence of this technique.

### 3.4. Waterwheel Plant Algorithm (WWPA)

In the space of potential solutions to the problem, the Waterwheel Plant Algorithm (WWPA) is a population-based approach that, via iteration, can give an adequate answer based on the search power of its population members. Each waterwheel comprising the Waterwheel Plant Algorithm (WWPA) population has its own values for the issue variables. This is because of the location that they hold in the search space. Accordingly, each waterwheel symbolizes a possible solution to the problem, which may be represented mathematically by a vector. The following matrix is used to depict the Waterwheel Plant Algorithm (WWPA) population, comprised of all the waterwheels. At the beginning of the Waterwheel Plant Algorithm (WWPA) implementation, the placements of the waterwheels in the search space are randomly adjusted using the following formula [59].

(3) $$P=\left[\begin{array}{c} P_{1}\\ \vdots \\ P_{i}\\ \vdots \\ P_{N} \end{array}\right]=\left[\begin{array}{ccccc} p_{1,1} & \cdots & p_{1,j} & \cdots & p_{1,m}\\ \vdots & \ddots & \vdots & ⋰ & \vdots \\ p_{i,1} & \cdots & p_{i,j} & \cdots & p_{i,m}\\ \vdots & ⋰ & \vdots & \ddots & \vdots \\ p_{N,1} & \cdots & p_{N,j} & \cdots & p_{N,M} \end{array}\right]$$

(4) $$p_{i,j}=lb_{j}+r_{i,j}.\left(ub_{j}-lb_{j}\right),i=1,2,...,N,j=1,2,...,m$$

where *N* is the number of variables and *m* is the number of waterwheels; $r_{i,j}$ is a random integer in the interval [0,1]; $lb_{j}$ and $ub_{j}$ are the lower and upper bounds of the j-th problem variable, respectively; *P* is the population matrix of waterwheel locations; $P_{i}$ is the i-th waterwheel, symbolizing a potential solution; and $p_{i,j}$ is the j-th dimension, representing the problem variable.

To ensure that the goal function can be computed for each waterwheel, each represents a potential solution. An appropriate representation of the values that have been decided to compose the objective function of the issue may be achieved through a vector, as demonstrated by example (3).

(5) $$F=\left[\begin{array}{c} F_{1}\\ \vdots \\ F_{i}\\ \vdots \\ F_{N} \end{array}\right]=\left[\begin{array}{c} F(X_{1})\\ \vdots \\ F(X_{i})\\ \vdots \\ F(X_{N}) \end{array}\right]$$

In this equation, *F* represents a vector that contains all of the values of the objective function, and $F_{i}$ represents the estimated value for the i-th waterwheel. The evaluations of the objective functions are the most important measure to identify the most effective solutions. Because of this, the most significant value of the objective function relates to the best candidate solution (also known as the best member), while the lowest value corresponds to the worst candidate solution (also known as the worst member). Because the waterwheels travel over the search area at different speeds in each iteration, the best response must change with time.

Waterwheels can locate the origin of pests because of their keen sense of smell, which makes them powerful predators. When the waterwheel begins to attack an insect, it does so whenever the bug enters its area of attack. After locating the insect, it launches an assault and hunts for it. To represent the first step of its population update process, the Waterwheel Plant Algorithm (WWPA) simulates the behavior of waterwheels described above. By modeling the waterwheel attack on the insect, which causes significant alterations in the location of the waterwheel in the search space, the exploration capability of Waterwheel Plant Algorithm (WWPA) is boosted, allowing it to perform better in discovering the optimal zone and escaping from local optimality. Using the following equation in connection with the simulation of the waterwheel’s approach to the insect, one may calculate the new location of the waterwheel with the help of this equation. If shifting the waterwheel to this place increases the value of the objective function, then the previous site will be switched out for the one mentioned further down in this paragraph.

(6) $$\vec{W}={\vec{r}}_{1}.\left(\vec{P}\left(t\right)+2K\right)$$

(7) $$\vec{P}\left(t+1\right)=\vec{P}\left(t\right)+\vec{W}.\left(2K+{\vec{r}}_{2}\right)$$

Alternately, if the answer does not improve after three iterations in a row, the waterwheel’s location can be altered by employing the equation presented below.

(8) $$\vec{P}\left(t+1\right)=Gaussian\left(\mu_{P},\sigma \right)+{\vec{r}}_{1}\left(\frac{\vec{P}\left(t\right)+2K}{\vec{W}}\right)$$

The random variables ${\vec{r}}_{1}$ and ${\vec{r}}_{1}$ have values that fall within the ranges of [0,2] and [0,1], respectively and accordingly. Furthermore, the variable *K* is an exponential variable that may take on values within the [0,1] range. The vector $\vec{W}$ represents the circumference of the circle in which the waterwheel plant will seek locations that have the potential to yield positive results.

An insect is captured using a waterwheel plant, and then it is transported to a feeding tube after being transported. A simulation of the behavior of waterwheels serves as the basis for the second stage of the population update process in Waterwheel Plant Algorithm (WWPA). Since the model of transporting the insect to the appropriate tube leads to the creation of small changes in the position of the waterwheel in the search space, the Waterwheel Plant Algorithm (WWPA)’s exploitation power is increased during the local search, and better solutions are converged near the ones that have already been discovered. This is made possible by the fact that the creators of Waterwheel Plant Algorithm (WWPA) first chose a new random location for each waterwheel in the population. This location is designed to be a “good position for consuming insects”, representing the natural action of waterwheels. As a result, the waterwheel is relocated to the new position if the value of the objective function is more significant at this new location, as demonstrated by the equations presented below.

(9) $$\vec{W}={\vec{r}}_{3}.\left(K{\vec{P}}_{best}\left(t\right)+r_{3}\vec{P}\left(t\right)\right)$$

(10) $$\vec{P}\left(t+1\right)=\vec{P}\left(t\right)+K\vec{W}$$

It is important to note that $\vec{P}\left(t\right)$ represents the current solution at iteration *t*, while ${\vec{P}}_{best}$ represents the best solution. The random variable ${\vec{r}}_{3}$ has values that fall between the [0,2] range. The following modification is implemented in the same manner as the exploration phase to guarantee that local minima are avoided. This occurs if the solution does not improve after three rounds.

(11) $$\vec{P}\left(t+1\right)=\left({\vec{r}}_{1}+K\right)\sin\left(\frac{F}{C}\theta \right)$$

In this context, the variables *F* and *C* are random variables with values that fall within the [−5,5] range. Furthermore, taking into account the following equation, the value of *K* drops in an exponential fashion:

(12) $$K=\left(1+\frac{2*t^{2}}{T_{max}}+F\right)$$

### 3.5. Particle Swarm Optimization Algorithm (PSO)

The Particle Swarm Optimization (PSO) technique is designed to simulate the intelligence of bird swarms in nature. This is accomplished by allowing particles (potential solutions) to be flown across the search area for issues. Velocity is the term used to describe the change in position that a particle experiences. The location of the particles shifts together over time. Equations (13) and (14) are used to update the velocity of a particle to a neighborhood best solution while it is in flight.

(13) $$v_{k+1}^{i}=v_{k}^{i}+c_{1}r_{1}\left(Pbest_{k}^{i}+x_{k}\right)+c_{2}r_{2}\left(gbest-x_{k}^{i}\right)$$

(14) $$x_{k+1}^{i}=x_{k}^{i}+v_{k+1}^{i}$$

The velocity of a particle is stochastically accelerated to its prior best location during the flight procedure. The location vector, $x_{i}$, and the velocity vector, $v_{i}$, are the two vectors that define a particle, *i*.

### 3.6. Comparison of WWPA with State-Of-The-Art Optimizers

A comparative evaluation of the Waterwheel Plant Algorithm (WWPA) and state-of-the-art (SOTA) metaheuristic optimization algorithms suitable for feature selection required analysis to validate its use in our hybrid method. The optimization framework consists of five algorithms, namely, the Genetic Algorithm (GA), Particle Swarm Optimization (PSO), and Grey Wolf Optimizer (GWO) with the Whale Optimization Algorithm (WOA) and Firefly Algorithm (FA). The adaptive characteristics of water plants serve as inspiration for WWPA, which controls its global search activity against its local optimization abilities. WWPA surpasses GA and PSO through dynamics agent position regulation by using environment-responsive movement rules, which make it different from traditional techniques. The algorithm maintains powerful search abilities through its mechanisms, which protect it from ending up in sub-optimal solutions.

WWPA differs from GWO and WOA since it omits the requirement of hierarchical structures and encircling regulation for natural behavior simulation. WWPA enables better flexibility for navigating complex and high-dimensional search regions in medical feature selection problems. The local exploitation strengths of FA and PSO produce slower convergence speeds when applied independently to sparse datasets that have either redundant or noisy features.

WWPAPSO represents a combined algorithm that utilizes WWPA for worldwide discovery and PSO for performing rapid convergence by exploiting local areas. The combination structure works best for binary feature selection because it respects diversity while enhancing solution quality steadily.

### 3.7. The Proposed WWPAPSO

Locating global optimal solutions to a problem is a complex undertaking. The technique that is being offered includes the presentation of two practical algorithms. Individuals move following their global and local best positions in the Waterwheel Plant Algorithm (WWPA), which is the first available algorithm. In contrast to the local best position, which refers to the best position that an individual has discovered up to this point, the global best position is the best position of the whole population. Due to this social behavior, people in the Waterwheel Plant Algorithm (WWPA) can converge on their global goal. Our suggested hybrid optimizer is a Waterwheel Plant Algorithm (WWPA), which we chose because of its robustness, dependability, and ease of use.

Our selection of WWPA occurred because it excels at exploring complex high-dimensional spaces similar to those found in cervical cancer feature data. The WWPA methodology maintains solution diversity and avoids premature convergence because of its advantages, which are ideal for biomedical feature selection in applications that face overlapping and noisy features.

The PSO algorithm is the second optimizer we have presented for our hybrid method. Swarm Optimization, often known as PSO, is a metaheuristic optimizer based on swarms and mimics particles’ social hierarchy and foraging behavior. Particles’ positions affect the individuals that are shifted within the PSO.

We chose PSO as an additional algorithm since it brings powerful exploitation capabilities. WWPA uses an exhaustive search, while PSO brings efficiency through global population best and accuracy through individual local best to optimize the algorithm’s search capabilities. The hybridization scheme explores wide-ranging search areas while performing local refinement, vital for selecting optimal medical data feature subsets.

**Figure 4:**
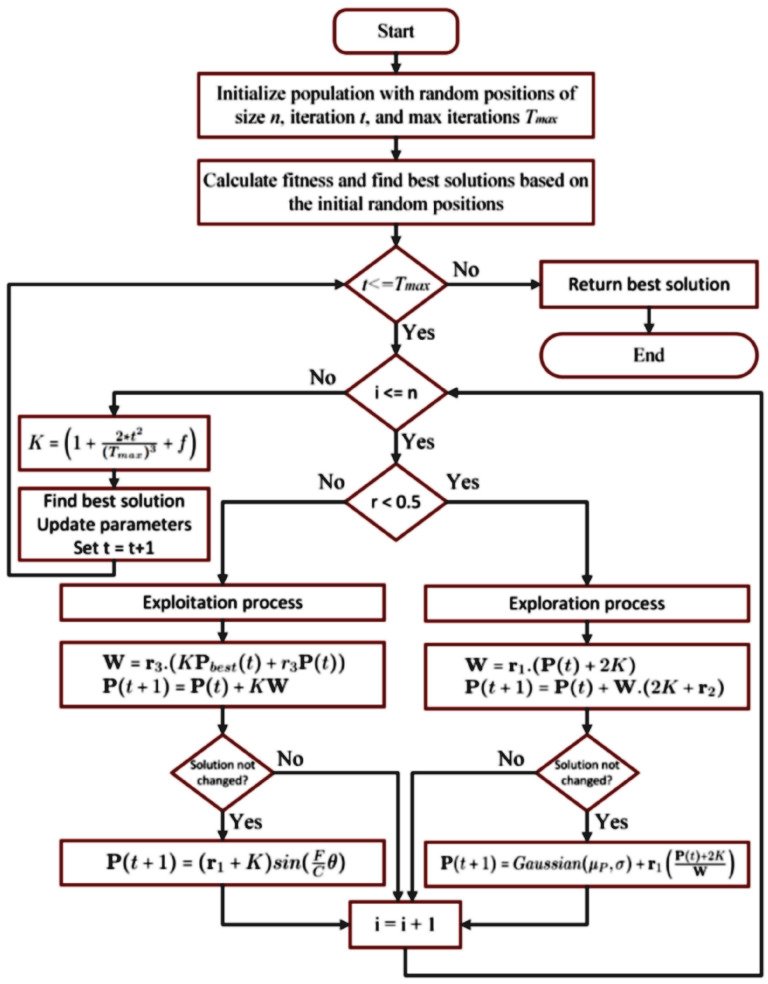
The steps of the proposed WWPAPSO algorithm.

The steps of the proposed WWPAPSO algorithm are depicted in Figure 4.

In Figure 4, the proposed WWPAPSO algorithm presents its workflow in illustrating how WWPA and PSO unite to execute hybrid feature selection and optimize classification accuracy. The method starts with populating a pool of candidate solutions that use binary forms to represent possible feature subsets as individual particles. The population spreads its members randomly throughout their defined search region boundaries. The following step applies a fitness evaluation using an objective function that derives classification accuracy from each particle’s Multilayer Perceptron (MLP) classifier. The fitness function rates how well a chosen set of features contributes to precise cervical cancer diagnosis. In WWPA, any waterwheel agent moves to new positions through dynamic equations intended for environmental interaction and exponential decay of adjustments. PSO provides exploitation through its ability to update particles by using their personal best positions together with the global best positions. A few iterations without noticeable progress will activate local search refinements with stochastic operators (Gaussian distribution or sinusoidal perturbation) for escaping local optima while diversifying the search. When the optimization process satisfies a termination condition (such as maximum iteration count or stagnation limits), the best-performing feature subset will be used to optimize an MLP for final classification purposes. Integrating the Waterwheel Plant Algorithm (WWPA) and Particle Swarm Optimization (PSO) produces a hybrid method that simultaneously accelerates feature subset selection and optimizes their quality because it capitalizes on their distinct beneficial characteristics for achieving accurate cervical cancer predictions.

## 4. Experimental Results

In this section, the results of the experiments are presented and explained. The section starts with the evaluation metrics, followed by the feature selection results, and then, finally, the classification results are presented and discussed.

### 4.1. Evaluation Metrics

When it comes to the field of medicine, verifying that a person is healthy is less important than making a correct diagnosis of a person who is suffering from a disease. For this reason, accuracy should not be the sole statistic examined when evaluating a model. F-measure, precision, specificity, sensitivity, and accuracy are the five performance measures that were utilized in this study to evaluate the effectiveness of our model concerning performance. There is a confusion matrix that is used to compute the metrics. The symbols TP, TN, FP, and FN make up a confusion matrix. This is a table structure that displays the performance of the model in a visual format. When a malignant individual is accurately predicted to have cancer, this is called a true positive (TP). TN stands for “true negative”, which refers to a person who does not have cancer but is also properly predicted to be cancer-free. When a person who does not have cancer is incorrectly identified as having cancer, this is referred to as a false positive (FP). FN is an abbreviation for “false negative”, which suggests that a person who has cancer is not affected by the disease. That FN is the most crucial component that has to be minimized to the greatest extent feasible is an abundantly evident reality. Following their fundamental notations, these metrics are defined as follows:

- Out of the total number of predictions, the model’s accuracy is described by Equation (15). Accuracy is the number of forecasts that are right.
  (15) $$Accuracy=\frac{TP+TN}{TP+TN+FP+FN}$$
- A model’s sensitivity may be defined as its capacity to identify individuals diagnosed with cervical cancer accurately. A sensitivity of one gives the impression that the model accurately predicted every individual who was diagnosed with cervical cancer. Equation (16) uses mathematics to define it in a mathematical sense.
  (16) $$Sensitivity=\frac{TP}{TP+FN}$$
- The algorithm’s capacity to accurately predict individuals who do not have cervical cancer is evaluated based on this parameter, which represents specificity. The definition of specificity may be found in Equation (17).
  (17) $$Specificity=\frac{TN}{FP+TN}$$
- The term “precision” refers to the proportion of individuals who have been diagnosed with cervical cancer and who have been accurately identified by the model as having the disease. Precision is a measurement of the proportion of individuals diagnosed with cervical cancer who agree with the model’s predictions. Equation (18) uses mathematics to define it in a mathematical sense.
  (18) $$Precision=\frac{TP}{TP+FP}$$
- F-Measure is the model’s precision and sensitivity harmonic mean. It combines the model’s accuracy and sensitivity, indicating its performance. If the F-measure is improved, then the number of individuals who have been incorrectly categorized as having or not having cervical cancer will be reduced. An explanation of what the F-Measure is may be found in Equation (19).
  (19) $$F-Score=\frac{TP}{TP+\frac{1}{2}\left(FP+FN\right)}$$

### 4.2. Feature Selection Results

The results obtained from the feature selection methods, presented in Table 3, for classifying cervical cancer using the binary Waterwheel Plant Algorithm and Particle Swarm Optimization (bWWPAPSO) showcase varying performances across multiple evaluation metrics. These metrics provide crucial insights into the efficacy and suitability of each method in identifying pertinent features for accurate classification. The average error rate, a pivotal metric indicating classification accuracy, reveals notable differences among the methods. Notably, the bWWPAPSO method stands out with the lowest average error rate of 0.712, suggesting a superior ability to accurately classify cervical cancer cases compared to other techniques such as bPSO, bBA, bWAO, bBBO, and others. This lower error rate implies higher precision in distinguishing between cancerous and non-cancerous cases, signifying the potential effectiveness of bWWPAPSO in this specific context. Considering the average select size, which measures the number of features selected by each method, bWWPAPSO demonstrates a relatively lower average select size of 0.685. A smaller average select size indicates a more concise feature subset, which can potentially reduce computational complexity and overfitting. However, this needs careful consideration alongside classification accuracy to strike an optimal balance between a reduced feature count and maintaining high prediction performance. The fitness metrics—average, best, worst, and standard deviation—provide further insights into the selected feature subsets’ quality, variability, and stability across different runs. Regarding average and best fitness, bWWPAPSO consistently displays competitive results, indicating its effectiveness in generating feature subsets with strong classification capabilities. Moreover, the relatively lower standard deviation (Std Fitness) observed in bWWPAPSO runs suggests a more stable performance than other methods like bBBO, bMVO, and others.

The proposed binary Waterwheel Plant Algorithm and Particle Swarm Optimization (bWWPAPSO) method exhibits promising results in accurately classifying cervical cancer, showcased through its lower average error rate and competitive fitness metrics. These findings suggest the potential effectiveness of bWWPAPSO in selecting a concise yet powerful subset of features crucial for classification. Nonetheless, additional validation studies, including testing on independent datasets and further analysis, are essential to affirm its robustness, generalizability, and suitability for real-world applications in cervical cancer classification.

The statistical analysis of feature selection results, presented in Table 4, for classifying cervical cancer using the binary Waterwheel Plant Algorithm and Particle Swarm Optimization (bWWPAPSO) presents a comprehensive overview of various statistical measures across different feature selection methods. The analysis encompasses descriptive statistics such as standard deviation, maximum, minimum, quartiles, mean, and other key parameters for ten feature selection methods. These statistics offer insights into the distribution, variability, and central tendencies of the performance metrics evaluated. Across the methods, the statistics unveil nuanced differences in the performance metrics. For instance, the minimum and maximum values indicate the range within which the performance metrics fluctuate. The minimum values showcase the least optimal performance achieved by each method, while the maximum values highlight the best-performing scenarios. In this case, the bWWPAPSO method demonstrates a minimum value of 0.710 and a maximum of 0.713, showcasing a relatively smaller range (0.003) than other methods, such as bBA, with a range of 0.026. The quartiles—25th, 50th (median), and 75th percentiles—indicate the spread of data around the median. Interestingly, the quartiles exhibit identical values for most methods, including bWWPAPSO, suggesting a consistent distribution of performance metrics within these methods. Moreover, the mean and standard deviation provide insights into the central tendency and dispersion of the data, respectively. The mean values for bWWPAPSO and other methods help gauge the average performance. At the same time, the standard deviation offers a measure of the variability or spread of the performance metric values around the mean. Here, bWWPAPSO displays a smaller standard deviation (0.001), indicating less variability in its performance across different runs than other methods. The statistical analysis of feature selection results showcases nuanced differences among various methods, providing valuable insights into their performance characteristics. The bWWPAPSO method demonstrates competitive and consistent performance, as indicated by its relatively smaller range, consistent quartile values, and lower standard deviation. However, while these statistics offer a quantitative understanding of performance, further analysis and validation, considering the trade-offs between accuracy, feature subset size, and stability, are essential to determine the most compelling feature selection method for cervical cancer classification.

The ANOVA (Analysis of Variance) test, presented in Table 5, applied to the feature selection results for classifying cervical cancer using the binary Waterwheel Plant Algorithm and Particle Swarm Optimization (bWWPAPSO) provides critical insights into the significance of differences among the performance metrics obtained from various feature selection methods. The ANOVA (Analysis of Variance) table consists of three main components: treatment, residual, and total, each revealing specific information regarding the variability and significance of the performance of the feature selection methods. Treatment: This section of the ANOVA table assesses the variance between the different treatment groups, i.e., the various feature selection methods. The Sum of Squares (SS) for treatment is 0.056, indicating the total variability attributed to differences among the methods. The Degrees of Freedom (DF) is 9, representing the number of feature selection methods minus one. The Mean Square (MS), calculated as SS divided by DF, is 0.0062. The F-statistic (F) measures the variance ratio between the methods to the variance within the methods. In this case, the F-statistic is 286.3, with degrees of freedom for the numerator (DFn) as nine and denominator (DFd) as 90, resulting in a highly significant *p*-value (*p* < 0.0001). This implies significant differences among the feature selection methods regarding their impact on classification performance for cervical cancer. Residual: This section of the table focuses on the variance within each method or the variability that the treatment cannot explain (feature selection methods). The SS for the residual is 0.002, indicating the unexplained variance within the methods. The DF is 90, representing the total number of observations minus the total number of treatment groups. The MS for the residual is 0.00002. Total: The total variability in the dataset is accounted for in this section. The Total SS is 0.058, encompassing the variance due to the treatment (feature selection methods) and the residual variance. The Total DF is 99, representing the sum of DF for treatment and residual. The ANOVA test results suggest significant differences among the feature selection methods (treatments) concerning their impact on the classification performance for cervical cancer. The highly significant *p*-value (*p* < 0.0001) indicates that the variability in performance metrics across these methods is not due to random chance. Still, instead, there are genuine differences in their effectiveness. This analysis underscores the importance of selecting the most appropriate feature selection method as it significantly influences the classification outcome in cervical cancer analysis.

The evaluation of feature selection methods through ANOVA analysis required additional experiments that ran the evaluations with different configurations to guarantee the robustness and reliability of results. The study examined the F-statistic and significance levels when changing DF values through evaluations for 20 and 30 repetitions for each method.

Table 6 and Table 7 present the ANOVA results for both configurations. The treatment design included a constant value of DF set to 9, representing the ten feature selection methods under analysis. The number of repetitions per method affected the residual DF value to be 190 for twenty runs and 290 for thirty runs. The statistical results display significant high values (*p* < 0.0001) across all tests, which validates the dependable nature of the feature selection evaluation.

The Wilcoxon signed-rank test, presented in Table 8, applied to the feature selection results for classifying cervical cancer using the binary Waterwheel Plant Algorithm and Particle Swarm Optimization (bWWPAPSO) aims to ascertain whether there are statistically significant differences between the performance of these methods. The test involves comparing measurements from the same dataset to determine if one method consistently outperforms the other. In this case, the theoretical median (expected performance) for all methods is 0, while the actual median performance of each method is given. The Wilcoxon signed-rank test examines the hypothesis that there is no difference in the medians of the paired samples. The “sum of signed ranks” (W), “sum of positive ranks”, and “sum of negative ranks” are calculated from the differences between the paired observations’ ranks. Here, the sum of signed ranks (W) for each method is 55, suggesting a consistent trend in performance across the methods evaluated. The “*p*-value (two-tailed)” associated with each method is 0.002, indicating a high significance level. This *p*-value suggests that there is only a 0.2% probability (assuming the null hypothesis is true) of observing such extreme differences in medians between the methods by chance alone. Hence, a low *p*-value leads to rejecting the null hypothesis, suggesting significant differences in performance between these feature selection methods for classifying cervical cancer. The "Discrepancy" column illustrates the difference between the theoretical and actual medians for each method, reflecting the magnitude of deviation from the expected performance.

**Figure 5:**
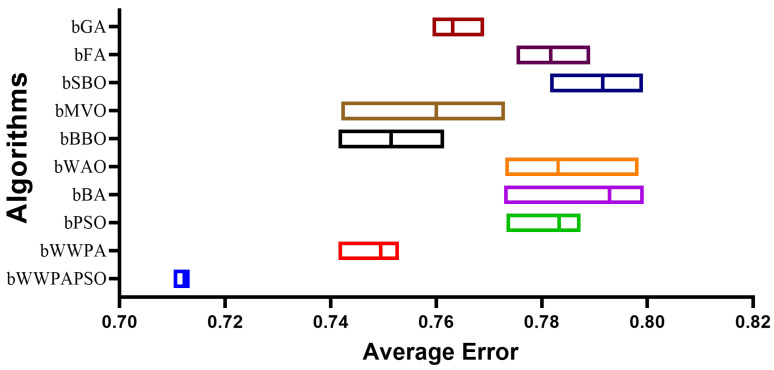
The average classification error of cervical using the proposed feature selection method.

The Wilcoxon signed-rank test reveals statistically significant differences in the performance of feature selection methods, highlighting that these methods do not perform equally when applied to cervical cancer classification. It signifies the importance of choosing the most effective method based on statistical significance and actual performance metrics when selecting features for classifying cervical cancer cases. On the other hand, the average classification error of cervical using the proposed feature selection compared to the other feature selection algorithms is shown in Figure 5. In this figure, it is clearly shown that the proposed feature selection algorithm achieves the lowest average error when compared to the other feature selection methods.

### 4.3. Cervical Classification Results

The classification results, shown in Table 9, for cervical cancer based on selected features demonstrate varying performances across different machine learning algorithms. These metrics, including Accuracy, Sensitivity (True Positive Rate—TPR), Specificity (True Negative Rate—TNR), Positive Predictive Value (PPV), Negative Predictive Value (NPV), and F-score, provide comprehensive insights into the effectiveness of each algorithm in correctly classifying cancerous and non-cancerous cases. The Neural Network (Multi-Layer Perceptron—MLP) exhibits the highest Accuracy among the models, achieving 0.881, indicating the proportion of correctly classified cases. Additionally, it showcases a high Sensitivity (TPR) of 0.862, demonstrating the model’s ability to correctly identify most of the positive (cancerous) cases. Its high Specificity (TNR) of 0.889 implies its effectiveness in accurately identifying negative (non-cancerous) cases. Moreover, the Neural Network achieves a substantial Positive Predictive Value (PPV) of 0.769 and an impressive Negative Predictive Value (NPV) of 0.938, emphasizing its ability to precisely predict positive and negative cases, respectively. The F-score of 0.813 highlights a balanced performance between precision and recall.

**Figure 6:**
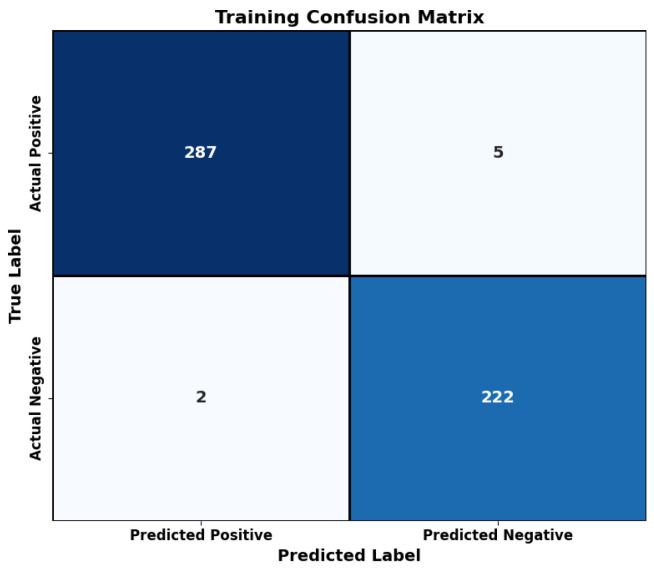
Confusion matrix of the training dataset showing classification results in terms of true/false positives and negatives.

The training data classification performance of the proposed model is depicted through its confusion matrix (Figure 6). The data visualization includes an informative table of true-positive and negative cases with false-positive cases and false-negative cases showing sensitivity and specificity numbers during training processes.

**Figure 7:**
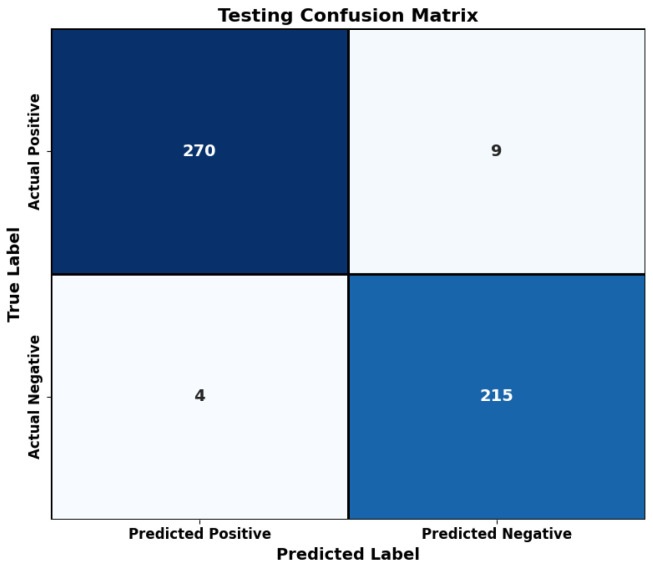
Confusion matrix of the test dataset showing classification outcomes.

A confusion matrix appeared in Figure 7 for a test of the proposed model’s generalization capability. The practical and stable performance of the model exists beyond training due to its near-perfect ratio of correct predictions and its minimal number of false outcomes.

The Random Forest algorithm demonstrates good overall performance with an Accuracy of 0.767. It achieves a notably high Sensitivity (TPR) of 0.896, indicating a robust capability to identify positive cases correctly. However, its Specificity (TNR) is comparatively lower at 0.537, suggesting a moderate ability to identify negative cases accurately. The Positive Predictive Value (PPV) and Negative Predictive Value (NPV) are 0.775 and 0.744, respectively. The F-score of 0.831 reflects a balanced performance between precision and recall. Other algorithms like Support Vector Machine, Gradient Boosting, K-Nearest Neighbors, Decision Tree, Logistic Regression, and AdaBoost exhibit varying levels of performance in terms of Accuracy, Sensitivity, Specificity, PPV, NPV, and F-score. They generally demonstrate moderate to good performance, with differences in their strengths in correctly classifying cervical cancer cases and their ability to avoid misclassification. The results highlight the diverse performance of machine learning algorithms in cervical cancer classification. While the Neural Network (MLP) and Random Forest show promising results with high Accuracy and balanced TPR-TNR trade-offs, the choice of the most suitable model should consider the specific needs of the application, balancing trade-offs between sensitivity, specificity, and predictive values for clinical or practical relevance.

The classification results, shown in Table 10, for cervical cancer based on selected features using the WWPAPSO+MLP method showcase outstanding performance across various evaluation metrics, highlighting its effectiveness in accurately distinguishing between cancerous and non-cancerous cases. This method combines the binary Waterwheel Plant Algorithm and Particle Swarm Optimization for feature selection, followed by classification using a Multi-Layer Perceptron (MLP) model. The WWPAPSO+MLP method achieves an exceptionally high Accuracy of 0.973, indicating the proportion of correctly classified cases among the total instances. Moreover, it demonstrates an impressive Sensitivity (TPR) of 0.988, suggesting an exceptional ability to identify positive (cancerous) cases correctly. Additionally, it maintains a strong Specificity (TNR) of 0.914, indicating its capability to identify negative (non-cancerous) cases accurately. The Positive Predictive Value (PPV) and Negative Predictive Value (NPV) are notably high at 0.978 and 0.952, respectively. This signifies the method’s ability to predict positive and negative cases precisely, emphasizing its reliability in making accurate predictions. The F-score of 0.983 showcases a balanced performance between precision and recall, indicating the WWPAPSO+MLP method’s ability to maintain high precision and recall simultaneously, making it a robust and well-rounded model for cervical cancer classification. Comparatively, other methods, including WWPA+MLP, PSO+MLP, WAO+MLP, FA+MLP, and GA+MLP, also exhibit strong performance in terms of Accuracy, Sensitivity, Specificity, PPV, NPV, and F-score. They demonstrate slightly lower metrics than the WWPAPSO+MLP approach but perform excellently in accurately classifying cervical cancer cases. The WWPAPSO+MLP method emerges as a highly accurate and reliable approach for cervical cancer classification based on selected features. Its exceptional performance across multiple evaluation metrics highlights its potential as a powerful tool in aiding accurate diagnosis and decision-making in clinical settings. However, while these results are promising, further validation studies and assessments on larger datasets are crucial to ensure their robustness and generalizability in real-world applications.

The ANOVA (Analysis of Variance) test, shown in Table 11, applied to the classification results of cervical cancer, assesses whether there are statistically significant differences in the performance among multiple treatment groups or classification methods. Treatment: This section examines the variation in classification performance attributed to the different treatment groups or methods. The Sum of Squares (SS) for treatment is 0.022, suggesting the total variability among the methods regarding their classification results. The Degrees of Freedom (DF) for treatment is 5, representing the number of treatment groups minus one. The Mean Square (MS), calculated as SS divided by DF, is 0.004472. The F-statistic (F) measures the variance ratio between the methods to the variance within the methods. In this case, the F-statistic is 227.8, with degrees of freedom for the numerator (DFn) as five and the denominator (DFd) as 54, resulting in a highly significant *p*-value (*p* < 0.0001). This indicates significant differences among the classification methods regarding their performance on cervical cancer classification. Residual: This table section assesses the unexplained variance or variability within each method, not accounted for by the treatment (classification methods). The SS for the residual is 0.001, representing the unexplained variance within the methods. The DF for the residual is 54, indicating the total number of observations minus the total number of treatment groups. The MS for the residual is $1.96\times 10^{-5}$. Total: The total variability in the dataset, encompassing both the treatment and residual variability, is accounted for in this section. The Total SS is 0.023, with a Total DF of 59, representing the sum of DF for treatment and residual. The ANOVA (Analysis of Variance) test results indicate significant differences in the performance of various classification methods used for cervical cancer classification. The highly significant *p*-value (*p* < 0.0001) suggests that the observed variability in performance metrics among these methods is unlikely due to random chance, indicating genuine differences in effectiveness. This analysis underscores the importance of selecting the most appropriate classification method as it significantly influences the outcome in cervical cancer classification. Further exploration, validation, and comparison of these methods on larger datasets or different populations are essential for a comprehensive understanding of their effectiveness and generalizability.

ANOVA tests became the method to evaluate the consistency of the optimization model component by running several experiments. The study included six optimization algorithms through which 20-run and 30-run experiments were conducted. Table 12 and Table 13 present the results. The treatment DF is 5 because six optimization methods were assessed in each execution. The remainder of the degrees of freedom in the model depends on how often each method was repeated. Statistical significance remains strong since both F-statistic results remain high and *p*-values remain below 0.0001 regardless of run size.

The Wilcoxon signed-rank test, shown in Table 14, applied to the classification results of cervical cancer, assesses whether there are statistically significant differences in the performance among multiple classification approaches. This non-parametric test is particularly useful when data may not meet the assumptions of normality and aims to determine if one method consistently outperforms the others. The table presents results for different classification approaches, including WWPAPSO+MLP, WWPA+MLP, PSO+MLP, WAO+MLP, FA+MLP, and GA+MLP. The “Theoretical median” represents the expected median performance (0 in this case), while the “Actual median” indicates the observed median performance for each method. The Wilcoxon signed-rank test computes the sum of signed ranks (W) based on the differences between paired observations’ ranks, indicating the consistency and direction of differences between the methods. In this case, all methods yield a sum of signed ranks (W) of 55, suggesting a consistent trend in performance across the methods evaluated. The “*p*-value (two-tailed)” associated with each method is 0.002 for all cases. This low *p*-value indicates a high significance level, suggesting that there is only a 0.2%

**Figure 8:**
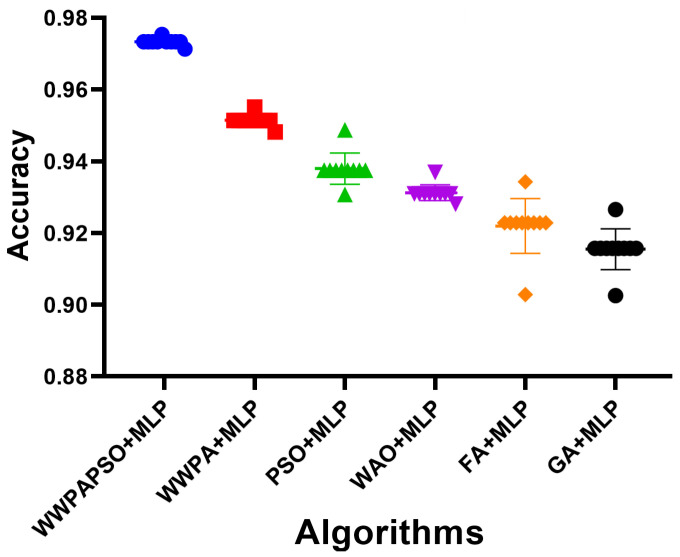
The accuracy of cervical classification using the proposed methodology.

On the other hand, Figure 8 presents the accuracy of the cervical cancer classification using the proposed approach in comparison to different approaches such as WWPA+MLP, PSO+MLP, WOA+MLP, FA+MLP, and GA+MLP. As shown in this figure, the classification achieved by the proposed approach outperforms the other approaches. This confirms the superiority of the proposed methodology.

**Figure 9:**
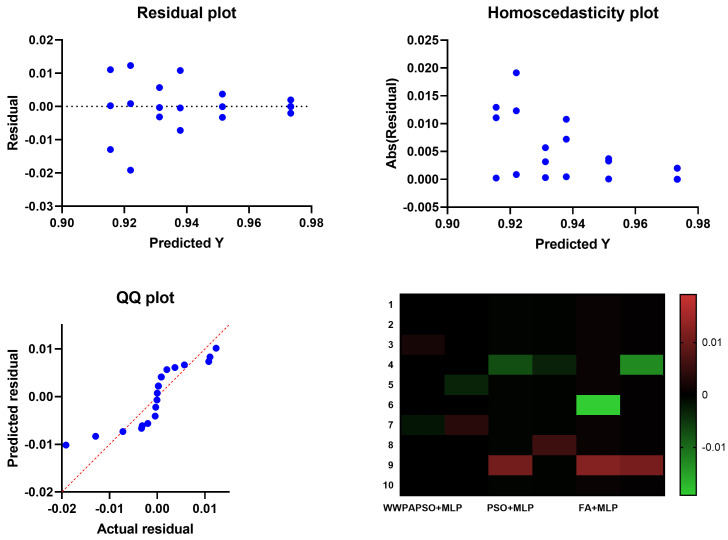
Visualizing the results of the ANOVA (Analysis of Variance) test based on the proposed methodology.

Moreover, the results of the ANOVA (Analysis of Variance) analysis are visualized in the plots shown in Figure 9. These plots include Residual, homoscedasticity, quartile-quartile (QQ), and heatmap plots. These plots show the proposed methodology’s effectiveness from the perspective of statistical analysis.

**Figure 10:**
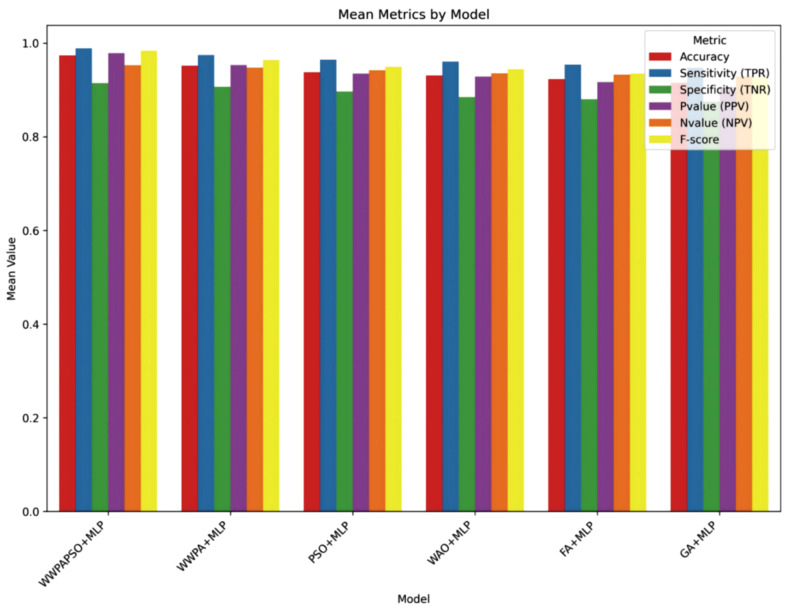
Mean values of performance metrics for various optimization algorithms with MLP.

Figure 10 illustrates the mean values of six performance metrics for different optimization algorithms combined with an MLP classifier. WWPAPSO+MLP achieves the highest mean values across most metrics.

**Figure 11:**
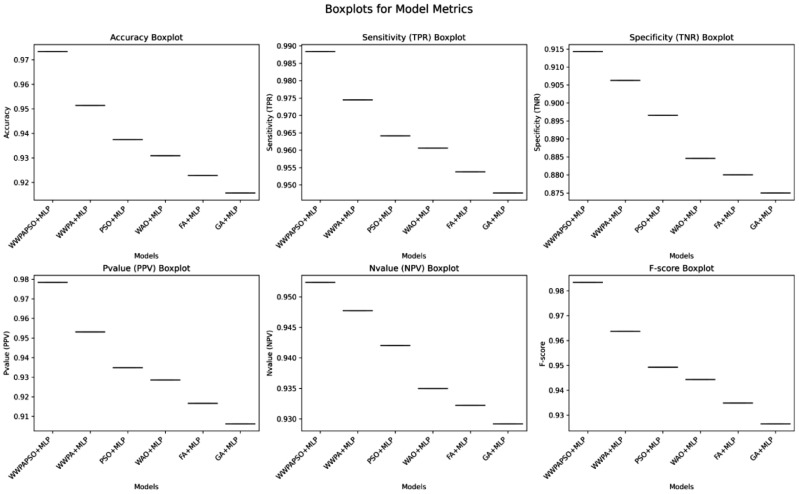
Boxplots comparing model metrics for various optimization algorithms combined with MLP classifiers.

Figure 11 presents boxplots comparing six key metrics across various optimization algorithms combined with an MLP classifier: WWPAPSO+MLP, WWPA+MLP, PSO+MLP, WAO+MLP, FA+MLP, and GA+MLP. The WWPAPSO+MLP model consistently outperforms others across all metrics, showcasing the highest median values for accuracy, sensitivity, specificity, and F-score, indicating its robustness and effectiveness in classification tasks.

**Figure 12:**
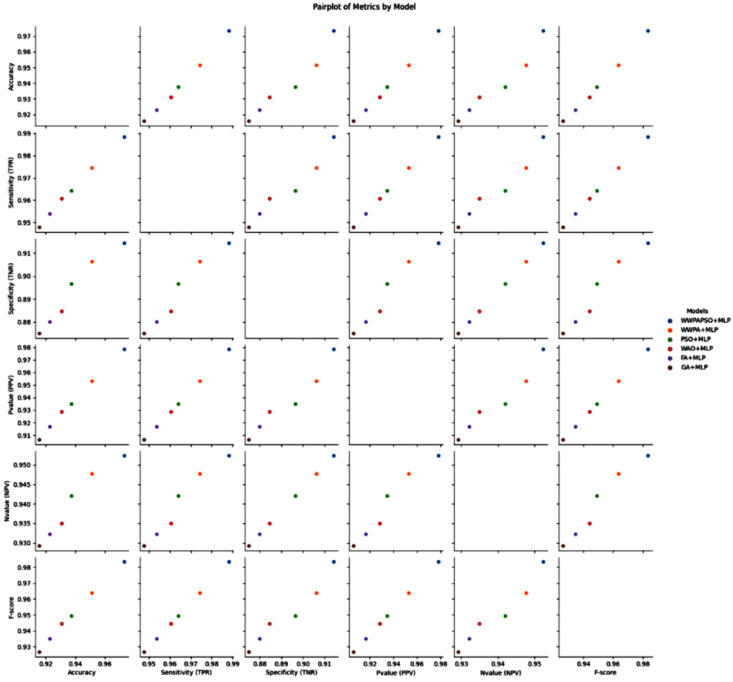
Pair plot visualizing relationships between performance metrics for optimization algorithms combined with MLP classifiers.

The pair plot in Figure 12 illustrates the relationships between performance metrics across multiple models. Each scatter plot in the grid compares a pair of metrics for all models, while diagonal elements display distributions of individual metrics. WWPAPSO+MLP consistently appears at the upper end of most metrics, showcasing its superior performance compared to the other models.

**Figure 13:**
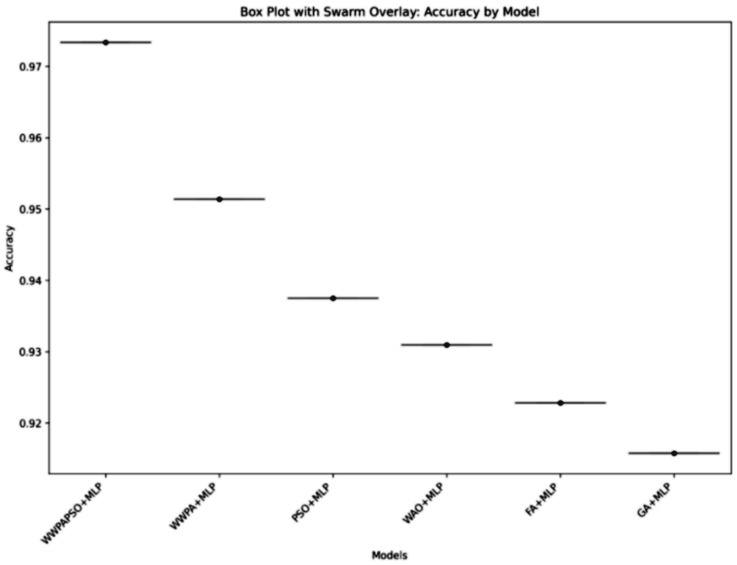
Box plot with swarm overlay visualizing the accuracy of various optimization algorithms with MLP.

Figure 13 displays the box plot with a swarm overlay. It highlights the accuracy distribution for each model while allowing for an examination of individual data points through the swarm overlay. WWPAPSO+MLP achieves the highest accuracy with minimal variance.

**Figure 14:**
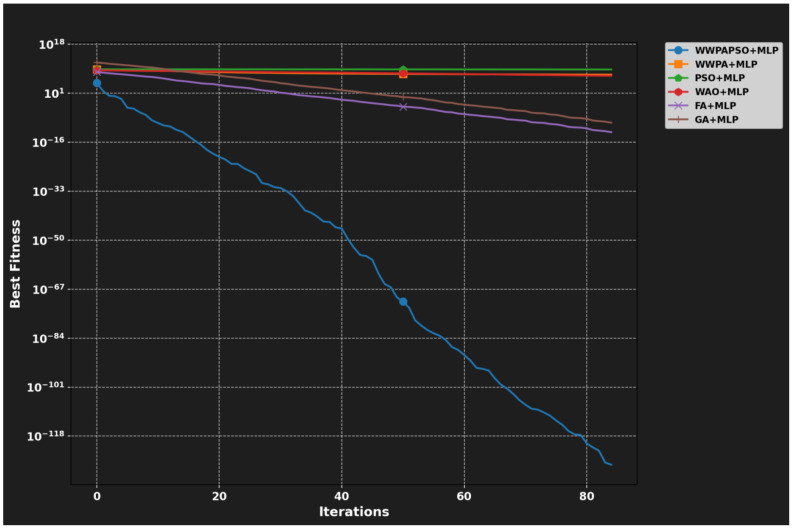
Convergence curve of WWPAPSO+MLP compared to other metaheuristics (WWPA, PSO, WOA, FA, an GA) with MLP. The Y-axis shows best fitness values in logarithmic scale over 85 iterations.

The proposed WWPAPSO technique has its convergence performance examined by matching it with stand-alone algorithms like the WWPA, PSO, GA, FA, and WOA, which utilize the same MLP classifier for fairness evaluation. Figure 14 shows the WWPAPSO+MLP combination reaching the optimal solution much faster and achieving deeper levels throughout the iterations than other optimizers that show limited or stagnant fitness improvements. The Meilleure performance of this hybrid approach demonstrates its effectiveness in avoiding trapped solutions by helping it achieve faster convergence, thus making it suitable for biomedical applications with complex datasets.

**Figure 15:**
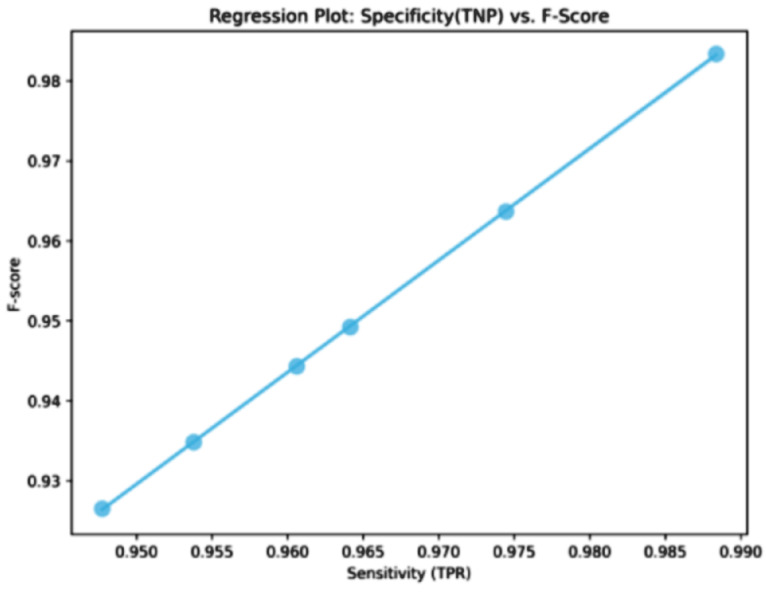
Regression plot showing the relationship between Sensitivity (TPR) and F-score across test runs.

The model classification consistency was evaluated through a regression analysis between Sensitivity (True-Positive Rate) and F-score across various configuration sets. Figure 15 demonstrates that both measurements show a positive relationship that indicates sensitivity improvements directly produce F-score improvements. The model performs excellently because it scans positive incidents effectively while balancing its precision score and recall statistics. The model alignment proves vital in medical diagnosis because it controls both missed cases and incorrect alerts with substantial risks.

**Figure 16:**
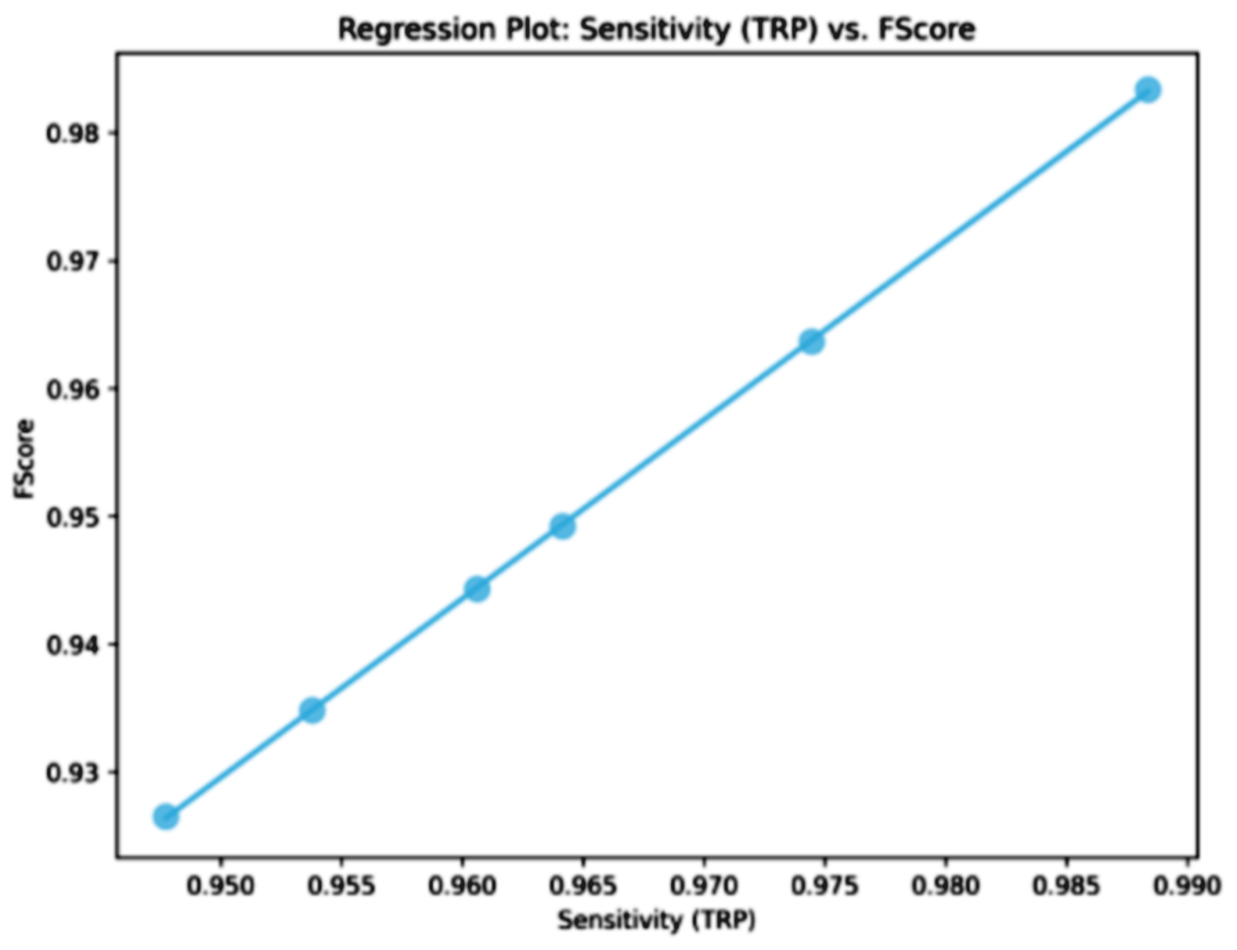
Regression plot showing the relationship between Sensitivity (TPR) and F-score across model evaluations.

We performed a regression analysis between sensitivity (True-Positive Rate) and F-score throughout multiple evaluations to study their correlation. The linear representation in Figure 16 demonstrates that elevated sensitivity directly improves the F-score. This evidence shows that the model successfully detects positive cases and sustains appropriate precision–recall ratios during medical diagnostics to reduce false-negative results.

**Figure 17:**
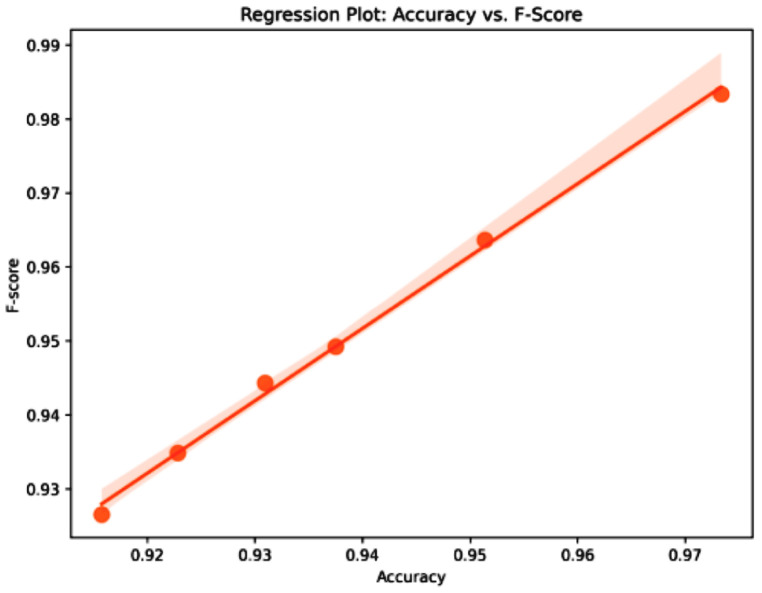
Regression plot showing the relationship between Accuracy and F-score across evaluation runs.

A regression analysis of overall accuracy and F-score was used for validation of model reliability across different evaluation trials. As demonstrated in Figure 17, a close linear correlation exists between classification accuracy and F-score. This indicates that higher accuracy measurements directly lead to better F-score results. The model demonstrates consistent performance, confirming that it identifies samples correctly and optimally balances precision and recall achievement.

**Figure 18:**
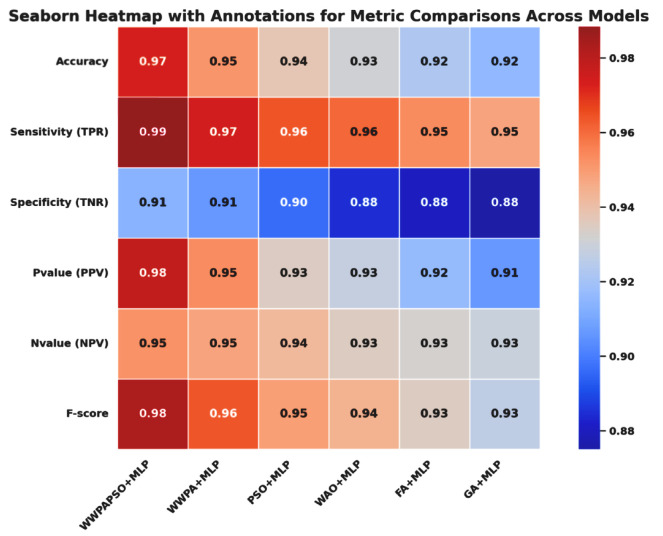
Heatmap of performance metrics across hybrid optimization models (Optimizer + MLP).

The paper uses heatmap visualization to provide detailed evaluation metric comparisons for optimization algorithms. The table presented in Figure 18 includes six hybrid models that merge a metaheuristic optimizer with MLP classifier and their corresponding performance metrics of Accuracy, Sensitivity (TPR), Specificity (TNR), Positive Predictive Value (PPV), Negative Predictive Value (NPV), and F-score. The developed WWPAPSO+MLP system demonstrates superior performance in all assessment indicators, including sensitivity, demonstrating its effective true identification capability with maintained precision. A gradient color scheme strengthens performance gap identification so users can easily evaluate their assessment models.

**Figure 19:**
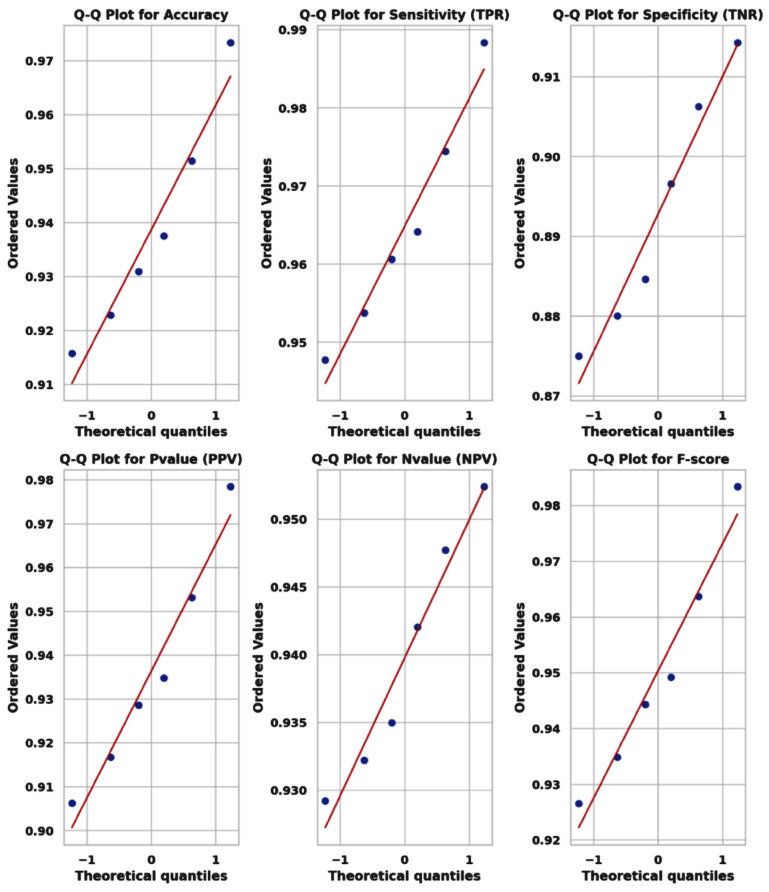
Q–Q plots for evaluating normality across classification metrics. Each subplot tests the distributional assumption for a specific metric.

The performance metric distribution normality analysis used Quantile–Quantile (Q–Q) plots across all optimization models. Each box in Figure 19 presents the results of Q–Q analysis performed on different evaluation metrics such as Accuracy and Sensitivity (TPR) and Specificity (TNR), and Positive Predictive Value (PPV), as well as Negative Predictive Value (NPV) and F-score. The distribution characteristics indicated by points next to the red diagonal line show normalcy for our performance metric data, thus validating parametric testing in our evaluation. Our comparative evaluation process achieves additional validation through this step because it strengthens the statistical foundation of our analysis.

The entire experimental workflow consisting of data processing, feature extraction model building, and assessment took place through Python 3.10. The development included the utilization of NumPy and Pandas libraries for data processing along with scikit-learn libraries for machine learning functions and preprocessing methods Matplotlib 3.9.0 and Seaborn libraries for graphics and TensorFlow/Keras libraries for MLP classification. A Dell Precision 3660 High-Performance Workstation functioned as the platform for experiments where it ran on a 12th Generation Intel Core i7-12700 processor with 2.10 GHz base frequency up to 4.9 GHz boost clock speed and 64 GB DDR5 RAM and a 2 TB SSD for quick data processing. A total of 858 records in the initial cervical cancer dataset comprised 36 features, but after data preprocessing, the research retained 737 complete records. The data collection was divided into two subset groups using an 80:20 percentage split method for training and validation purposes.

## 5. Conclusions

As one of the leading causes of premature death among women is cervical cancer, this paper proposed a novel methodology for early classification of this type of cancer. A new feature selection algorithm and a new classification technique are the foundations of the unique strategy that we present in this research for the early classification of cervical cancer. A blend of the Waterwheel Plant Algorithm and the Particle Swarm Optimization algorithm is the foundation of the new feature selection algorithm, which is denoted by the acronym bWWPAPSO. Meanwhile, the new classification approach is referred to as WWPAPSO+MLP. This method considers the optimization of the parameters of a multilayer perceptron neural network. The suggested method is tested using a dataset accessible to the general public to determine whether or not it is successful. This dataset was preprocessed and balanced with SMOTETomek, which utilized both undersampling and oversampling techniques. This was carried out since the dataset was imbalanced and included missing values. By means of a comparative examination of the suggested technique that has been carried out, it has been proven that the effectiveness of feature selection and class imbalance based on the classifier’s accuracy, sensitivity, and specificity may be demonstrated. With a sensitivity of 98.8% and an accuracy of 97.3%, the approach that has been suggested reaches unparalleled performance. On the other hand, several statistical tests were carried out to validate the statistical significance and distinction of the approach provided. The Wilcoxon test and the Analysis of Variance (ANOVA) are included in these tests. The outcomes of these tests are consistent with what was anticipated to occur due to the proposed strategy. It is advised that more research be carried out using a larger dataset. This will allow for more in-depth analysis and comprehension to be carried out, as well as the creation of a more accurate classification model for the same issue. For the long-term management of cervical cancer in clinical and customized medical management by decision support system, it is planned that further study will be carried out in the future.

### Future Work and Limitations

The proposed system showcases effective performance in cervical cancer prediction by combining bWWPAPSO for feature selection and MLP as a deep learning classifier while facing multiple limitations. Despite its widespread usage, the experimental evaluation uses only the UCI cervical cancer dataset, but the data contains missing values and an unbalanced class distribution. Future researchers should test the model using larger clinical data from multiple healthcare facilities to demonstrate universal applicability across varied population types and setting environments. The system conducts all training and assessment tasks within a unified central computing setting. Implementing this model under distributed or federated learning settings would improve data protection and security, particularly when working with clinical information that requires distributed storage. Further analysis should focus on developing privacy-preserving network designs because of their natural applicability. The model utilizes SMOTETomek for class balance correction, yet better techniques such as adaptive resampling and cost-sensitive learning would probably boost outcomes primarily for scarce positive instances. The proposed bWWPAPSO algorithm received empirical validation but did not undergo direct comparison with all existing bio-inspired feature selection methods introduced since its development. Future work should expand the benchmarking phase to include other hybrid and metaheuristic optimization tools, specifically focusing on medical data applications. External medical teams face challenges when using deep learning systems in healthcare because these systems remain difficult to explain. Implementing XAI methods, including SHAP and LIME, for model decision interpretation will enhance medical practitioner trust levels in future research.

## 6. Additional Information

Correspondence and requests for materials should be addressed to S.K.T. and M.M.

## Figures and Tables

**Table 1:** Summary of feature selection methods and studies.

Method	Refs.	Contributions
Embedded	[25,26]	LASSO introduced for parameter estimation and feature selection based on penalized least squares regression using the L1-penalty function.
	[27]	Applied LASSO for cardiovascular disorder prediction; achieved 97.65% accuracy using Random Forest Bagging Method (RFBM) compared to 92.65% without LASSO.
	[28]	LASSO effectively identified key attributes for predicting spot prices of energy, reducing mean prediction error by 16.9%.
	[29]	Embedded feature selection with LASSO pruned unnecessary features in neural networks, creating group sparsity and improving model performance.
	[23,24]	Hybrid embedded approaches combining filter and wrapper methods for computational efficiency and improved accuracy.
Wrapper	[30,31]	Recursive Feature Elimination (RFE) removes weak features recursively, resulting in better prediction models with reduced dimensionality.
	[32]	Demonstrated reduced runtime with fewer features using RFE.
	[33,34]	Used RFE for gene selection in microarray data, significantly reducing thousands of features.
	[35]	Compared RFE with Sequential Feature Selection (SFS); highlighted differences in complexity and use cases.
	[36]	Applied RFE for cervical cancer prediction, achieving 91.04% accuracy, 91.94% specificity, and 89% AUC using SVM.
Filter	[21]	Statistical analysis used for selecting features based on correlation with the target variable.
	[37]	Filter techniques are fast and computationally efficient but lack interaction between features and model dependence.
	[2]	Illustrated the importance of feature selection in creating effective models by comparing various techniques like Boruta, SA, and RFE for cervical cancer risk factor detection.

**Table 2:** Summary of studies on cervical cancer classification.

Ref.	Methodology	Results	Limitations	Dataset
[11]	Decision Tree (DT) classifier applied to the Risk Factors dataset	Demonstrated classification capability without feature selection.	No feature optimization was applied, leading to suboptimal results.	Feature (structured numerical data)
[47]	SVM-based methods on the Risk Factors dataset	Evaluated three SVM methods with moderate classification success.	Overfitting not addressed; no preprocessing to improve model robustness.	Feature (structured numerical data)
[9]	Gaussian Naive Bayes (GNB), DT, Logistic Regression (LR), KNN, SVM	DT achieved the highest accuracy (97%) among the classifiers.	Overfitting due to hold-out validation on a small dataset; better validation techniques like k-fold were suggested.	Feature (structured numerical data)
[8]	GBM, SVM, RF combined with SMOTE, Genetic Algorithm (GA), Bayesian Optimization	GBM had the highest sensitivity (77.8%), outperforming other models.	Sensitivity could be further improved; feature optimization was not fully addressed.	Feature (structured numerical data)
[10]	DT classifier without addressing class imbalance	Demonstrated decision-making capability for classification tasks.	Class imbalance skewed decision boundaries; no preprocessing applied to balance the dataset.	Feature (structured numerical data)
[12]	Boosted Decision Trees, Decision Forests, Decision Jungle with SMOTE	Boosted Decision Tree achieved the highest AUC (97%).	Did not use feature selection to reduce dimensionality; redundant features may affect performance.	Feature (structured numerical data)
[50]	RF with SMOTE, RFE, and PCA for feature reduction	Achieved 96% accuracy with RF; combined feature reduction techniques demonstrated effectiveness.	Limited exploration of other ML models for comparison.	Feature (structured numerical data)
[51]	SVM, XGBoost, RF with SMOTE	XGBoost and RF outperformed SVM in sensitivity (94% and 95%, respectively).	Did not investigate additional optimization techniques or ensemble methods for improvement.	Feature (structured numerical data)
[52]	Voting classifier (DT, LR, RF) with SMOTE and PCA	Improved accuracy, sensitivity, and AUC significantly; addressed dimensionality reduction and class imbalance.	SMOTE caused larger, less specific decision areas, affecting classification precision.	Feature (structured numerical data)
[54]	Ensemble methods with a voting mechanism	Improved classification performance; addressed challenges in prior studies.	Sensitivity remained low due to unbalanced datasets.	Feature (structured numerical data)
[55]	CNN-ELM applied to the Herlev database	Achieved 99.5% accuracy for two-class classification and 91.2% for seven-class classification.	High computational cost; neural networks lack interpretability and require large datasets.	Image data (medical imaging data)
[57]	RF with SMOTETomek, DBSCAN, and iForest for outlier detection	Successfully handled class imbalance and outliers; achieved robust predictions with RF.	Limited comparison with other classifiers; potential over-reliance on preprocessing methods.	Feature (structured numerical data)
[13]	ML-based survival prediction	Emphasized survival analysis; extended ML use from diagnosis to prognosis.	Did not focus on classification tasks; lacks performance benchmarking.	Feature (structured numerical data)
[14]	RF, DT, SVM, KNN, Gradient Boosting, AdaBoost	Achieved 100% classification accuracy with several models; included a public perception survey.	Did not address class imbalance or feature selection; lacks external dataset validation.	Feature (structured numerical data)
[15]	Federated ML with IoMT, fuzzed neuron alignment, and blockchain	Achieved high accuracy (99.26%) with secure and privacy-preserving model training.	High complexity and overhead; lacks interpretability and model transparency.	Feature (structured numerical data)
[16]	Combined ML (SVM, GradBoost, DT, KNN, RF, XGBoost)	Achieved 96% accuracy and 98% precision, recall, and F1-score; promoted hybrid classifiers.	No mention of privacy or statistical significance testing; limited real-world deployment analysis.	Feature (structured numerical data)
[58]	Optimized Deep CNN with augmentation and cross-validation	Developed a robust skin cancer classification system with high accuracy on dermoscopic images.	Focused on skin cancer only; does not consider federated learning, privacy, or feature selection.	Image data (dermoscopic)

**Table 3:** The results of variance feature selection methods.

	bWWPAPSO	bWWPA	bPSO	bBA	bWAO	bBBO	bMVO	bSBO	bFA	bGA
Avg error	0.712	0.749	0.783	0.793	0.783	0.751	0.760	0.792	0.782	0.763
Avg Select size	0.685	0.885	0.885	1.024	1.048	1.049	0.982	1.055	0.920	0.827
Avg Fitness	0.795	0.812	0.810	0.833	0.818	0.816	0.840	0.850	0.862	0.823
Best Fitness	0.697	0.732	0.790	0.723	0.782	0.805	0.765	0.793	0.781	0.726
Worst Fitness	0.796	0.799	0.858	0.824	0.858	0.892	0.883	0.873	0.878	0.841
Std Fitness	0.618	0.622	0.622	0.632	0.624	0.667	0.673	0.683	0.659	0.624

**Table 4:** Statistical analysis of the feature selection results.

	bWWPAPSO	bWWPA	bPSO	bBA	bWAO	bBBO	bMVO	bSBO	bFA	bGA
Number of values	10	10	10	10	10	10	10	10	10	10
Minimum	0.710	0.742	0.773	0.773	0.773	0.742	0.742	0.782	0.775	0.759
25% Percentile	0.712	0.750	0.783	0.793	0.783	0.752	0.760	0.792	0.782	0.763
Median	0.712	0.750	0.783	0.793	0.783	0.752	0.760	0.792	0.782	0.763
75% Percentile	0.712	0.750	0.783	0.793	0.783	0.752	0.760	0.792	0.782	0.763
Maximum	0.713	0.753	0.787	0.799	0.798	0.762	0.773	0.799	0.789	0.769
Range	0.003	0.011	0.014	0.026	0.025	0.020	0.031	0.018	0.014	0.010
Mean	0.712	0.749	0.783	0.792	0.783	0.752	0.760	0.791	0.782	0.763
Std. Deviation	0.001	0.003	0.004	0.007	0.006	0.005	0.007	0.004	0.003	0.002
Std. Error of Mean	0.000	0.001	0.001	0.002	0.002	0.001	0.002	0.001	0.001	0.001
Sum	7.122	7.490	7.827	7.915	7.834	7.515	7.595	7.913	7.818	7.633

**Table 5:** The analysis of variance (ANOVA) test results when applied to the feature selection results.

	SS	DF	MS	F (DFn, DFd)	*p*-Value
Treatment	0.056	9	0.0062	F (9, 90) = 286.3	*p* < 0.0001
Residual	0.002	90	0.00002		
Total	0.058	99			

**Table 6:** ANOVA test results using 20 evaluation runs for feature selection methods.

	SS	DF	MS	F (DFn, DFd)	*p*-Value
Treatment	0.1082	9	0.01203	F (9, 190) = 369.9	*p* < 0.0001
Residual	0.006178	190	0.00003251		
Total	0.1144	199			

**Table 7:** ANOVA test results using 30 evaluation runs for feature selection methods.

	SS	DF	MS	F (DFn, DFd)	*p*-Value
Treatment	0.1640	9	0.01822	F (9, 290) = 529.1	*p* < 0.0001
Residual	0.009987	290	0.00003444		
Total	0.1740	299			

**Table 8:** The Wilcoxon signed rank test results when applied to the feature selection results.

	bWWPAPSO	bWWPA	bPSO	bBA	bWAO	bBBO	bMVO	bSBO	bFA	bGA
Theoretical median	0	0	0	0	0	0	0	0	0	0
Actual median	0.712	0.750	0.783	0.793	0.783	0.752	0.760	0.792	0.782	0.763
Number of values	10	10	10	10	10	10	10	10	10	10
Sum of signed ranks (W)	55	55	55	55	55	55	55	55	55	55
Sum of positive ranks	55	55	55	55	55	55	55	55	55	55
Sum of negative ranks	0	0	0	0	0	0	0	0	0	0
*p*-value (two-tailed)	0.002	0.002	0.002	0.002	0.002	0.002	0.002	0.002	0.002	0.002
Discrepancy	0.712	0.750	0.783	0.793	0.783	0.752	0.760	0.792	0.782	0.763

**Table 9:** The classification results achieved by variance machine learning models.

	Accuracy	Sensitivity (TPR)	Specificity (TNR)	*p* Value (PPV)	N Value (NPV)	F-Score
Neural Network (MLP)	0.881	0.862	0.889	0.769	0.938	0.813
Random Forest	0.767	0.896	0.537	0.775	0.744	0.831
Support Vector Machine	0.753	0.865	0.556	0.776	0.698	0.818
Gradient Boosting	0.753	0.885	0.519	0.766	0.718	0.821
K-Nearest Neighbors	0.747	0.865	0.537	0.769	0.690	0.814
Decision Tree	0.740	0.885	0.481	0.752	0.703	0.813
Logistic Regression	0.740	0.844	0.556	0.771	0.667	0.806
AdaBoost	0.740	0.885	0.481	0.752	0.703	0.813

**Table 10:** The classification results achieved by optimization of the MLP model.

	Accuracy	Sensitivity (TPR)	Specificity (TNR)	*p* Value (PPV)	N Value (NPV)	F-Score
WWPAPSO+MLP	0.973	0.988	0.914	0.978	0.952	0.983
WWPA+MLP	0.951	0.974	0.906	0.953	0.948	0.964
PSO+MLP	0.938	0.964	0.897	0.935	0.942	0.949
WAO+MLP	0.931	0.961	0.885	0.929	0.935	0.944
FA+MLP	0.923	0.954	0.880	0.917	0.932	0.935
GA+MLP	0.916	0.948	0.875	0.906	0.929	0.927

**Table 11:** The analysis of variance (ANOVA) test results applied to the classification results.

	SS	DF	MS	F (DFn, DFd)	*p*-Value
Treatment	0.022	5	0.004472	F (5, 54) = 227.8	*p* < 0.0001
Residual	0.001	54	$1.96\times 10^{-5}$		
Total	0.023	59			

**Table 12:** ANOVA test results using 20 evaluation runs for optimization algorithms.

	SS	DF	MS	F (DFn, DFd)	*p*-Value
Treatment	0.0443	5	0.00886	F (5, 114) = 510.8	*p* < 0.0001
Residual	0.001977	114	0.00001734		
Total	0.04628	119			

**Table 13:** ANOVA test results using 30 evaluation runs for optimization algorithms.

	SS	DF	MS	F (DFn, DFd)	*p*-Value
Treatment	0.06636	5	0.01327	F (5, 174) = 610.9	*p* < 0.0001
Residual	0.00378	174	0.00002173		
Total	0.07014	179			

**Table 14:** The Wilcoxon test results applied to the classification results.

	WWPAPSO+MLP	WWPA+MLP	PSO+MLP	WAO+MLP	FA+MLP	GA+MLP
Theoretical median	0	0	0	0	0	0
Actual median	0.973	0.951	0.938	0.931	0.923	0.916
Number of values	10	10	10	10	10	10
Sum of signed ranks (W)	55	55	55	55	55	55
Sum of positive ranks	55	55	55	55	55	55
Sum of negative ranks	0	0	0	0	0	0
*p*-value (two-tailed)	0.002	0.002	0.002	0.002	0.002	0.002
Discrepancy	0.973	0.951	0.938	0.931	0.923	0.916

## Data Availability

The data that support the findings of this study are available at https://archive.ics.uci.edu/dataset/383/cervical+cancer+risk+factors, accessed on 12 December 2024.
